# Probiotic pasta consumption improves lipid metabolism and reduces gut permeability in overweight and obese adults: a randomized controlled trial

**DOI:** 10.1016/j.crfs.2025.101215

**Published:** 2025-10-03

**Authors:** Silvia Tagliamonte, Roberta Barone Lumaga, Francesca De Filippis, Vincenzo Valentino, Maria Aponte, Raffaele Romano, Paola Vitaglione

**Affiliations:** a Department of Agricultural Sciences, University of Naples Federico II, 80055, Portici, Italy; b Task Force on Microbiome Studies, University of Naples Federico II, 80134, Naples, Italy

**Keywords:** Probiotic intervention, Gut permeability, Personalized nutrition, Colonization status, Lipid metabolism, Gut microbiome

## Abstract

This study investigates the effects of a *Shouchella clausii* UBBC-07 spore-enriched pasta (ProbP) compared to a conventional pasta (ConP) on metabolic health and gut microbiome composition in overweight and obese individuals. A 4-weeks randomized controlled trial was conducted with 40 participants (17 females and 23 males, aged 35.4 ± 2.0 years, body mass index 27.6 ± 0.4 kg/m^2^), all habitual pasta consumers. Participants were randomly assigned to replace their habitual pasta with 80 g/day of either ProbP (n = 20) or ConP (n = 20) while maintaining their regular diet. Blood, urine, and fecal samples were collected at baseline and after 4 weeks to assess changes in clinical variables and gut microbiome composition. Consumption of ProbP led to an 11 % increase in serum high-density lipoprotein (HDL) cholesterol and improved gut permeability compared to baseline. Gut microbiome analysis revealed that 50 % of ProbP consumers had increased *S. clausii* abundance in feces. These “responders” (R) exhibited distinct baseline microbiome traits and experienced reductions in body weight (−1 %, p = 0.03) and diastolic blood pressure (−6 %, p = 0.049) compared to Non-responders (NR) to *S. clausii* colonization. Additionally, R showed a 17 % reduction in the low-density lipoprotein (LDL)-to-HDL ratio (p = 0.02) compared to baseline, correlated with increased fecal levels of *Akkermansia muciniphila* (p = 0.054) and *Harryflintia acetispora*, alongside reductions in *Ruminococcus torques* and *Dorea longicatena*. In conclusion, ProbP consumption improved blood lipid metabolism, likely through enhanced gut barrier function. The baseline gut microbiome influenced probiotic colonization ability, providing greater health benefits for a subset of participants.

## Highlights

• Probiotic pasta improved HDL levels and gut barrier in overweight individuals.
• *S. clausii* colonization occurred in 50 % of participants after 4 weeks of intake.
• Responders to colonization showed reductions in weight and diastolic pressure.
• Baseline microbiome traits affected probiotic colonization and metabolic response.

## Graphical abstract

**Figure:**
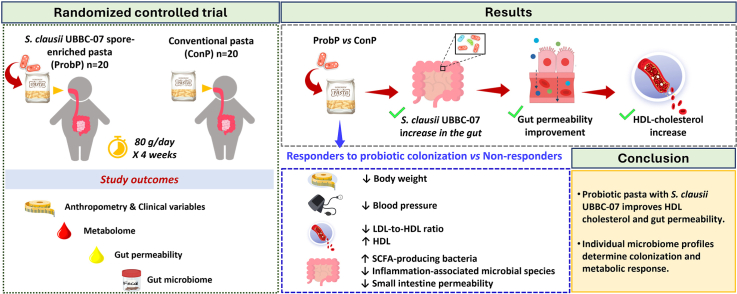

## 1 Introduction

The prevalence of overweight and obesity is a global health problem, contributing significantly to noncommunicable chronic diseases (NCD) such as cardiovascular disease, type 2 diabetes and certain types of cancer (Phelps et al., 2024). Sedentary lifestyle and nutritionally unbalanced diets are positively associated with the risk of obesity and NCD (Milanlouei et al., 2020; O'Hearn et al., 2023). Beyond genetics, the human gut microbiome is a key player in host's health as it can modulate various pathways involved in immunity, energy, lipid and glucose metabolism also through the interactions with the gut barrier functions (Zhang et al., 2024; Wu et al., 2024; Ross et al., 2024; Di Vincenzo et al., 2023). Dietary habits are key determinants of the composition and functionality of the gut microbiome (Cuevas-Sierra et al., 2019; Van Hul et al., 2024). Therefore, diet-induced microbiota shaping may be harnessed to induce changes in the host's physiology that prevent the onset of the NCD or retard their progression, through personalized nutrition and precision medicine approaches (Kolodziejczyk et al., 2019; De Filippis et al., 2018). In this context, probiotics have been widely acknowledged to sustain a healthy gut ecosystem (Anwer and Wei, 2024; Obayomi et al., 2024). This recognition, along with the rising consumer demand for functional foods (i.e. foods that provide health benefits beyond basic nutrition) have increased the food industry's interest in developing probiotic-enriched products in recent years (Obayomi et al., 2024).

The Food and Agriculture Organization of the United Nations (FAO) and the World Health Organization (WHO) guidelines recommend that probiotic products contain at least 10^6^ CFU per gram at the time of consumption to ensure a “minimum therapeutic” effect. Additionally, the strains must survive both the shelf life and passage through the upper intestinal tract to deliver their health benefits at the target site (Ramirez-;Olea et al., 2024). Spore-forming bacteria from the previous *Bacillus* genus [more recently re-classified into different genera (Patel and Gupta, 2020; Oren and Garrity, 2022)] are extensively utilized as probiotic microorganisms due to their ability to withstand various technological processes (such as heating, drying, and cooking), remaining alive after exposure to the low gastric pH (Blaiotta et al., 2023; Ghelardi et al., 2022), germinating in the small intestine and modulating the immunity of the host (Yu et al., 2019; Dang et al., 2024).

For instance, Angelino and colleagues showed that a 12-weeks consumption of whole-grain pasta, enriched with barley β-glucans and *Heyndrickxia coagulans* (formerly *Bacillus coagulans)* BC30,6086, decreased serum concentrations of hs-CRP and LDL/HDL cholesterol compared to a whole-grain pasta, in a subset of obese volunteers (Angelino et al., 2019). In addition, *Shouchella clausii* UBBC-07 (formerly *Bacillus clausii*) has been demonstrated to mitigate acute gastrointestinal symptoms in both children and adults through immunomodulation and inhibition of pathogens growth in the gastrointestinal tract (Sudha et al., 2013, 2019). To the best of our knowledge, no studies have investigated the metabolic and microbiome effects of *S. clausii* UBBC-07 delivered within a staple food matrix such as pasta.

We hypothesized that the consumption of pasta enriched with S. clausii UBBC-07 spores (ProbP) would beneficially modulate metabolism and gut microbiome composition, compared to conventional pasta (ConP), by improving gut barrier integrity and influencing lipid mediators, such as endocannabinoids (ECs) and N-acylethanolamines (NAEs), which are key regulators of appetite, energy metabolism, and host-microbiota interactions (Witkamp, 2018, Cani et al., 2016). To test this hypothesis, we conducted a 4-week randomized controlled trial in overweight and obese subjects, assessing fasting metabolic parameters, inflammatory status, gut permeability, appetite feelings, dietary intake, anthropometric measurements, and the circulating profiles of ECs and NAEs following consumption of ProbP compared to ConP.

## 2 Materials & methods

### 2.1 Chemicals and reagent+

Acetonitrile and water LC/MS grade were purchased from Carlo Erba, Milan, Italy; formic acid, the indoxyl sulfate (2642-37-7) and D-Mannitol ^13^C (132202-29-0) from Sigma-Aldrich, Milan, Italy. The lactulose (4618-18-2), mannitol (69-65-8) and sucralose (56038-13-2) were purchased from Farmalabor, Canosa di Puglia, Italy. The 2-arachidonoylglycerol (53847-30-6), arachidonoylethanolamide (94421-68-8), oleoylethanolamide (111-58-0), linoleoylethanolamide (68171-52-8), palmitoylethanolamide (544-31-0), and stearoylethanolamide (111-57-9) standards were purchased from Cayman Ann Arbor, USA.

### 2.2 Food products

The probiotic pasta was produced from a durum wheat flour added with the spores (10^7^ CFU/g) of the probiotic *Shouchella clausii* UBBC-07 strain (Blaiotta et al., 2023). Specifically, the pasta was produced using durum wheat semolina mixed with water in which the probiotic spores had been resuspended, resulting in a final dough water content of 42 %. The dough was then extruded into "penne" shape and subsequently dried, producing a standard dried pasta product. The probiotic spores were added during the mixing stage, prior to extrusion. Importantly, the concentration of spores remained stable throughout processing and was comparable at the time of consumption to the initial amount added to the dough. The ConP was made with the same durum wheat flour without the addition of the probiotic strain.

### 2.3 Study design and participants

We investigated the blood lipids, glucose and insulin, gut permeability, urinary indoxyl sulfate and gut microbiome in 40 overweight/obese subjects in response to a 4-weeks dietary intervention with ProbP or ConP. The study was conducted at the University of Naples Federico II and was approved by the Campania 3 Ethics Committee (Protocol number: 271/21). Each participant provided written informed consent and received no financial compensation for participation. The trial was registered at ClinicalTrials.gov (number NCT04962633) and ended when the last group of participants completed the protocol (study start date: January 2022; actual primary completion date: December 2022; study completion date: December 2022).

Serum lipids (including cholesterol and triglycerides), fasting blood glucose and, serum high sensitivity C-reactive protein (hs-CRP) were registered as primary outcomes on ClinicalTrials.gov (NCT04962633). Secondary outcomes included changes in urinary excretions of lactulose/mannitol ratio and sucralose following the gut permeability test (GPT), stool frequency, body composition, plasma endocannabinoids (ECs) and N-acylethanolamines (NAEs), gut microbiota composition, daily energy intake and appetite sensations.

**Fig. 1:**
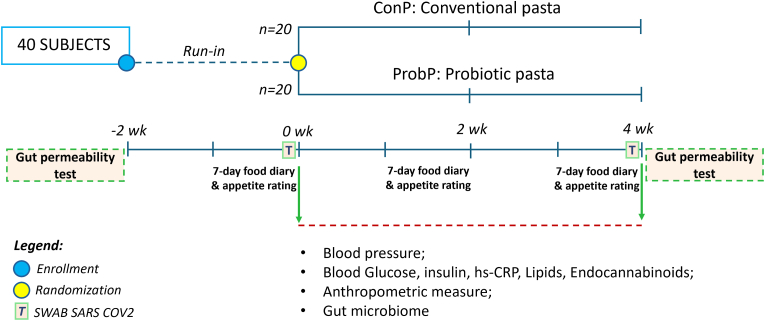
Study design.

Fig. 1 depicts the study design. Selected subjects were 18–60 years old; had a body mass index (BMI) above 25 kg/m^2^; were not pregnant, lactating, or taking medicines; did not have relevant organic, systemic or metabolic diseases; no history of abdominal surgeries, food intolerance or alcohol abuse; and did not consume probiotics, laxatives, or antibiotics within 3 months prior to enrolment. The subjects were further screened based on their habitual consumption of pasta (≥1 serving/day), fruit and vegetable intake (<3 servings/day), no probiotics and prebiotics consumption, whole-grain and/or fiber-enriched foods, and low physical activity level (≤500 metabolic equivalent min/week).

Forty subjects (17 females/23 males, average BMI 27.6 ± 0.4 kg/m^2^, age 35.4 ± 2.0 years) were enrolled and randomized to consume 80 g per day of ProbP (n = 20) or ConP (n = 20).

### 2.4 Dietary intervention

Participants underwent a 2-week run-in period prior to randomization, after which they were allocated to either the ProbP or ConP group based on a randomization sequence previously generated by a statistician using a computer-generated permuted block randomization scheme (n = 5). Both participants and outcome assessors were blinded to the intervention assignment.

Diet, physical activity level, quality of life, and fecal output were assessed by participants using a self-recorded 7-day food diary, the International Physical Activity Questionnaire (IPAQ) (Craig et al., 2003), the 12-item Short-Form Health Survey (SF-12) (Ware et al., 1996), and the King's Stool Chart (KSC) (Whelan et al., 2008), at baseline, and at 2 and 4 weeks. At the same time points, participants also rated their appetite using visual analogue scales before each daily meal and after dinner (Green et al., 1997). The self-recorded 7-d food diaries were analysed to assess daily energy and nutrient intakes using the software MetaDieta (METEDA S.r.l.). Anthropometric characterization of the participants consisted of body weight, height measurements and body composition estimation conventional bioelectrical impedance analysis with a single frequency 50-kHz bioelectrical impedance analyzer (BIA 101 RJL; Akern Bioresearch). Height of subjects was measured during the selection phase to the nearest 0.5 cm with a stadiometer (Model 213; Seca). Body weight was measured, after voiding, with subjects wearing light clothing to the nearest 0.1 kg on a digital scale (Model 703; Seca). Waist circumference was measured on undressed subjects at the midpoint between the lower margin of the last palpable rib and the top of the iliac crest. Hip circumference was measured around the widest portion of the buttocks, with the tape parallel to the floor.

At baseline, all participants were provided with either ProbP or ConP to be consumed daily. They were instructed to consume 80 g per day of the assigned product throughout the 4-week intervention. At baseline and after 4 weeks, following 10 h of fasting, participants visited the CDS San Ciro clinic (Portici), where blood samples were drawn and blood pressure was measured. Anthropometric measurements were then taken, and participants completed the SF-12, KSC, and IPAQ questionnaires. Blood samples were collected via venipuncture into serum separator tubes, EDTA-containing tubes, and EDTA tubes added with a protease inhibitor cocktail. The samples were centrifuged at 2400g per 10 min at 4 °C, and the supernatants were stored at – 80 °C prior to analysis. On the same day, participants also provided faecal samples for gut microbiome analysis.

### 2.5 Gut microbiome

The gut microbiome was analysed by shotgun metagenomics. Microbial DNA was extracted from faecal samples following the protocol reported by (Tagliamonte et al., 2023), in accordance with the International Human Microbiome Standard Consortium (IHMSC) SOP 07. DNA libraries were sequenced on the Illumina NovaSeq platform, generating 2x150bp, paired-end reads. Human reads were removed from metagenomics sequences using the Best Match Tagger (BMtagger; https://hmpdacc.org/hmp/doc/HumanSequenceRemoval_SOP.pdf) and the Human Sequence Removal pipeline developed within the Human Microbiome Project. Reads were quality-filtered using PRINSEQ 0.20.4 (Schmieder and Edwards, 2011), trimming bases with a Phred score <15 and discarding those <75 bp. High-quality reads were imported in MetaPhlAn 3.0 (Beghini et al., 2021) to obtain quantitative, species-level taxonomic profiles. For each sample, high-quality reads were assembled using MEGAHIT v. 1.2.2 (Li et al., 2015). Contigs <1000 bp were filtered out and genes were predicted using MetaGeneMark v. 3.26 (Zhu et al., 2010). Predicted genes were aligned against the Carbohydrates-Active enZYmes (CAZy) database (non-redundant at 90 % identity) by using DIAMOND v. 2.0.4 (Buchfink et al., 2015). An e-value cutoff of 1e^−5^ was applied, and a hit was required to display >90 % of identity over at least 75 % of the query length to be kept. The number of hits was normalized dividing by the total number of predicted genes in each sample. CAZy richness was expressed as the sum of the normalized counts of CAZy genes for each sample.

### 2.6 Gut permeability test

Gut barrier function was assessed using the GPT based on oral administration of a three-sugar probe and subsequent measurement of urinary excretion at defined time intervals (Li et al., 2016). The GPT was conducted two weeks prior to the start of the nutritional intervention and at the end of the study, approximately three days after the final visit. Two days before the test, participants were instructed to avoid milk, dairy products, and foods containing artificial sweeteners. On the day before testing, they consumed a standardized lunch and dinner consisting of boiled rice and roasted chicken or fish. On the test day, following an overnight fast, participants ingested 100 mL of a solution containing lactulose (5 g), mannitol (2 g) and sucralose (2 g), and to collected urine over 24 h into two separate containers. Urine collected from 0 to 5 h was used to assess small intestinal permeability, while urine collected from 5 to 24 h was used to evaluate colonic permeability. Urinary concentrations of lactulose, mannitol, and sucralose were quantified by LC-MS/MS.

### 2.7 Analysis of lactulose, mannitol and sucralose by LC-MS/MS

Lactulose, mannitol and sucralose concentrations in urine samples were quantified as described by (Tagliamonte et al., 2023). Urine was diluted 1:50 with acetonitrile/water (50:50) and centrifuged at 21100g × 10 min at 4 °C. Thereafter, supernatants were filtered and injected onto LC-MS/MS. Chromatographic separation was performed using an HPLC apparatus provided with two micropumps, PerkinElmer Series 200 (Norwalk, CT, USA). The compounds were separated on a TSKgel amide 80, 3 μm column (2 × 150 mm) (TOSOH BIOSCIENCE, Germany) with a setting temperature of 45 °C and a flow rate of 0.2 mL/min and the injection volume was 5 μL. Monitored compounds were separated by using a binary gradient mobile phase composed of mobile phase A (13 mM ammonium acetate in distilled water) and mobile phase B (50 % acetonitrile) and programmed as follows: 75 % B (2 min), 75–5 % B (6 min), 5 % B (8 min), 5–75 % B (12 min), constant 75 %B (15 min). The acquisition was performed in negative ion mode on an API 3000 triple quadrupole mass spectrometer (Applied Biosystems, Canada) equipped with a TurboIonSpray source in MRM (Multiple Reaction Monitoring). Calibration curves parameters are detailed in Supplementary Table 1.

### 2.8 Analysis of plasma endocannabinoids and N-acylethanolamines by LC-MS/MS

Circulating endocannabinoids (ECs) and N-acylethanolamines (NAEs) were measured, as these lipid mediators are key regulators of energy metabolism, inflammation, and gut-microbiota signalling (Witkamp, 2018). Plasma samples (500 μL) previously diluted 1:2 with distilled water were added with 50 μL of the internal standard 200 ng/mL solution of Arachidonoylethanolamide d8 (AEA d8) (Cayman Chemical, Ann Arbor, MI) as previously described (Tagliamonte et al., 2021). Then, the samples were vortexed and centrifuged 21,100g for 5 min at 4 °C. Oasis HLB cartridges (1 cc/30 mg Waters) were preconditioned with 1 mL of methanol and equilibrated using 1 mL of H2O. Samples were introduced onto the cartridges and were washed with 1 mL of aqueous methanol (40 %), and the monitored compounds were eluted in 1 mL of acetonitrile. The eluate was dried under nitrogen flow and reconstituted in 100 μL acetonitrile/water (50:50) before the LC–MS/MS analysis. The compounds were separated on a Synergi Max RP 80 column (50 × 2.1 mm) (Phenomenex, USA) with a setting temperature of 30 °C and a flow rate of 0.2 mL/min. The injection volume was 10 μL. The monitored compounds were eluted by a linear gradient of H_2_O and 0.2 % formic acid (solvent A) and CH_3_CN (solvent B). The eluting gradient was set as follows: 50–79 % B (10 min), 79–95 % B (1 min), constant at 95 % B (2 min) and finally returning to the initial conditions within 2 min. The acquisition was performed in positive ion mode on an API 3000 triple quadrupole mass spectrometer in MRM. Calibration curves parameters are detailed in Supplementary Table 2.

### 2.9 Clinical variables

Clinical blood variables, such as fasting serum glucose, lipids, and hs-CRP, insulin, and HOMA-index were measured according to the official methods of analysis. Specifically, glucose, cholesterol, and triglycerides were measured by enzymatic colorimetric methods (ABX Diagnostics, Roche Molecular Biochemicals, and Wako Chemicals GmbH) on a Cobas Mira autoanalyzer (ABX Diagnostics); hs-CRP was measured using a turbidimetric immunoassay (ADVIA, 1800; Siemens Healthineers); insulin concentration was measured by ELISA (DIAsource ImmunoAssays S.A.) on Triturus Analyzer (Diagnostics Grifols, S.A.); fasting insulin resistance was evaluated by the Homeostatic Model Assessment for Insulin Resistance [HOMA-IR = (fasting glucose, mmol/L)∗(fasting insulin, mU/L)/22.5] (Matthews et al., 1985).

### 2.10 Indoxyl sulfate analysis in urine by LC-MS/MS

Urine samples at baseline were analysed for indoxyl sulfate as previously reported (Tagliamonte et al., 2023). Tenfold diluted urine samples were centrifuged at 21100g × 10 min at 4 °C and filtered with regenerated cellulose membrane filters (0.2 μm) prior to LC-MS/MS analysis. The chromatographic separation was performed using an HPLC apparatus coupled to an API 3000 MS as already described above. The compounds were separated on a Kinetex 2.6 μ C18 100 Å column (100 mm × 2.1 mm) (Phenomenex, Torrance, CA) with setting temperature at 40 °C and eluted by a linear gradient of a water (0.1 % formic acid) (solvent A) and acetonitrile (0.1 % formic acid) (solvent B) with a flow rate of 200 μL/min and volume injection of 10 μL. The eluting gradient was adapted as follows: 5 % B from 0 to 0.5 min, 5–70 % B from 0.5 to 1.5 min, 70–95 % B from 1.5 to 3.5 min, 95 % B from 3.5 to 5 min, 95–5 % B from 5 to 6 min and kept at 5 % B until 11 min. Indoxyl sulfate showed a [M−H]^-^ ion at 212 *m*/*z*, and the daughter ion *m*/*z* 132 generated with collision energy (CE) of 33 V. Calibration curves were built in the linear range 1–15 μg/mL (y = 760.57x + 2068.6, R^2^ = 0.991).

### 2.11 Statistical analysis

The sample size was calculated considering the primary endpoints fasting serum hs-CRP, glucose and total cholesterol. According to a previous study, a sample size of 19 participants per group could detect a 30 % change in serum hs-CRP levels (Angelino et al., 2019). Whereas according to Meslier and colleagues (2020), 16 volunteers per group were considered adequate to detect significant changes in serum cholesterol and glucose levels (Meslier et al., 2020) with an *α*-error of 0.05, 80 % power, and 2-sided testing.

Statistical analysis and visualization were carried out in R version 4.0.3 (https://www.r-project.org). After variables were checked for normality, significantly skewed variables were natural-log transformed [ln(x + k), with k values zeroing the skewness]. Variables showing a normal distribution according to the Shapiro–Wilk test, an independent-samples *t*-test was performed to assess differences between groups. For non-parametric variables, the Mann–Whitney test was conducted to detect between-group differences. The repeated-measures ANOVA and Bonferroni adjustment for multiple comparisons to assess the effect of ProbP and ConP consumption on daily appetite sensations and self-reported habitual diet over the study period.

A multivariate regression model to identify those taxa significantly associated (q-value ≤0.05) with the ProbP and ConP consumption was performed by using MaAsLin2 (Mallick et al., 2021).

A linear discriminant analysis (LDA) effect size (LEfSE) was performed to explore the differentially abundant taxa at baseline among Responders and Non-responders participants within the ProbP group (Segata et al., 2011). To test the correlation among variation in gut microbiome, clinical and anthropometrical variables over the study period, pairwise Spearman's rank correlations within ProbP group. The chord diagram of correlation was visualized using the Hmisc and circlize packages. The heatmap showing the relative abundances of microbial genus was visualized using the Hmisc package and the function heatmap.2. The genus-level compositions were clustered using the partitioning around medoids (PAMs) algorithm (Arumugam et al., 2011). In order to explore the different response to ProbP consumption of clinical and gut microbial profiles among Responders and Non-responders participants, a Partial Least Squares Discriminant Analysis (PLS-DA, plsda function) was applied (library mixOmics) on anthropometrical, clinical and gut microbiome normalized datasets (scale function). The same package was employed for the integration of three datasets and functions using the DIABLO model (Data Integration Analysis for Biomarker discovery using Latent cOmponents) (Singh et al., 2019). Moreover, statistical significance of the distance between Responder and Non-responder groups in the co-inertia analysis was computed using the Hotelling T2 test (library Hotelling).

## 3 Results

### 3.1 Study participants and metabolic parameters

**Fig. 2:**
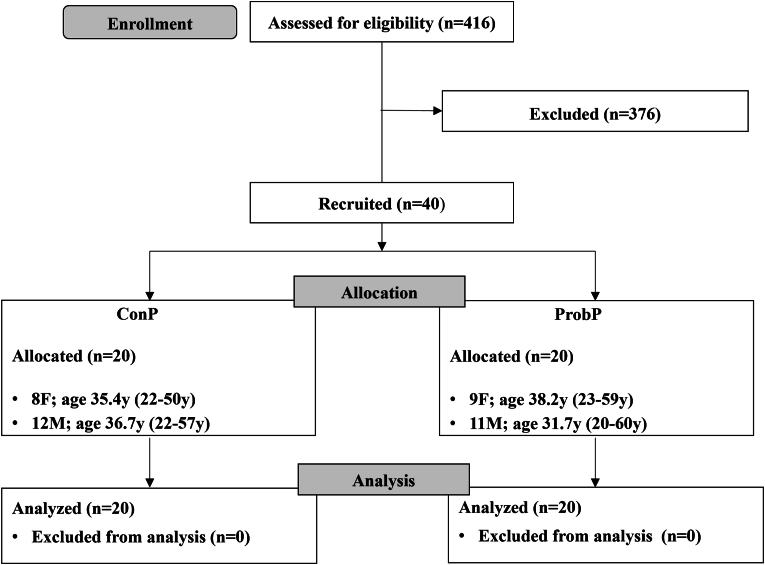
Participant flow diagram.

The participant flow is reported in Fig. 2.

Table 1 shows the general, anthropometric, lifestyle and psychological characteristics of the 20 (8F/12M, average BMI: 27.8 ± 0.5 kg/m^2^, age: 36.2 ± 2.5 years) participants who consumed ConP and 20 (9F/11M, average BMI: 27.5 ± 0.6 kg/m^2^, age: 34.7 ± 3.1 years) participants who consumed ProbP.

**Table 1:** General, anthropometric, lifestyle, and psychological characteristics of participants who consumed a conventional pasta (ConP) and probiotic pasta (ProbP) at baseline and after 4 weeks (4 wk).

	ConP		ProbP		p-values
	Baseline	4wk	Baseline	4wk	Δ_(4wk-baseline)_
**Sex (n, F/M)**	8/12		9/11		
**Age**	36.2 ± 2.5		34.7 ± 3.1		0.55
**Height (cm)**	170.5 ± 1.8		170.4 ± 2.0		0.94
**Body weight (kg)**	80.8 ± 2.1	81.0 ± 2.1	80.2 ± 2.9	80.0 ± 2.8	0.55
**BMI (kg/m^2^)**	27.8 ± 0.5	27.8 ± 0.5	27.5 ± 0.6	27.4 ± 0.6	0.52
**Waist circumference (cm)**	91.0 ± 2.1	91.0 ± 2.2	90.7 ± 2.9	89.8 ± 2.5	0.31
**Hip circumference (cm)**	105.5 ± 0.8	105.4 ± 1.1	105.5 ± 1.5	105.6 ± 1.5	0.32
**Waist/Hip**	0.9 ± 0.02	0.9 ± 0.02	0.9 ± 0.02	0.9 ± 0.02	0.28
**Body fat (%)**	26.9 ± 1.1	25.8 ± 1.1	27.8 ± 1.6	27.8 ± 1.5	0.17
**Body fat-free mass (%)**	54.0 ± 2.1	55.2 ± 2.1	52.4 ± 2.2	52.2 ± 2.1	0.09
**Systolic Blood Pressure (mmHg)**	119.3 ± 2.9	117.5 ± 2.9	115.2 ± 2.4	112.9 ± 2.7	0.81
**Diastolic Blood Pressure (mmHg)**	78 ± 2.0	76.8 ± 1.9	73.3 ± 1.8	71.6 ± 1.9	0.70
**Physical activity(MET min/week)**^a^	3261.0 ± 876.5	2939.5 ± 648.3	1690.1 ± 353.9	1747.1 ± 388.6	0.69
**King's stool chart score**	3.2 ± 0.2	3.3 ± 0.2	3.3 ± 0.2	3.3 ± 0.4	0.59
**Defecatory frequency in 24h (n)**	1.2 ± 0.1	1.2 ± 0.1	1.1 ± 0.1	1.1 ± 0.1	0.91

∗p < 0.05 within-group difference assessed by paired T-test. Pairwise time points (Δ) difference ConP *vs* ProbP were assessed by Independent-sample T-test or Mann-Whitney test depending on normal distribution of the data. Data are expressed as means ± SEM.

a MET: Metabolic equivalent of tasks.

The results of clinical variables monitored over the study and reported in Table 2, showed that ProbP consumption increased serum levels of HDL-cholesterol (p-value = 0.008) after the intervention vs baseline. The other clinical variables and circulating endocannabinoids and N-acylethanolamines were not affected by the intervention (Supplementary Fig. 1).

**Table 2:** Clinical variables monitored at baseline and after 4 week (4 wk) consumption of a conventional pasta (ConP) or probiotic pasta (ProbP).

	ConP		ProbP		p-values
	Baseline	4wk	Baseline	4wk	Δ_(4wk-baseline)_
**Glucose (mg/dL)**	92.7 ± 1.9	93.8 ± 1.8	92.0 ± 2.0	92.0 ± 1.6	0.66
**Insulin (μU/mL)**	8.3 ± 0.9	8.8 ± 0.9	7.9 ± 0.6	7.8 ± 0.8	0.52
**HOMA-index**	2.0 ± 0.2	2.1 ± 0.3	1.8 ± 0.2	1.8 ± 0.2	0.37
**Total cholesterol (mg/dL)**	159.3 ± 5.5	160 ± 7.8	148.8 ± 4.8	151.9 ± 3.7	0.78
**HDL-cholesterol (mg/dL)**	43.6 ± 2.3	46.1 ± 2.3	41.8 ± 1.9	46.45 ± 2.2**∗**	0.32
**LDL-cholesterol (mg/dL)**	98.9 ± 3.8	95.1 ± 3.8	89.4 ± 3.9	86.55 ± 3.1	0.85
**LDL-to-HDL ratio**	2.4 ± 0.1	2.2 ± 0.1**∗**	2.2 ± 0.2	2.0 ± 0.1∗	0.85
**Tryglicerides (mg/dL)**	83.8 ± 6.7	98.2 ± 6.7	88.5 ± 12.9	93.60 ± 11.2	0.70
**hs-CRP (mg/L)**	1.2 ± 0.2	1.1 ± 0.2	1.91 ± 0.6	1.7 ± 0.5	0.99

∗p < 0.05 within-group difference assessed by paired T-test. Pairwise time points (Δ) difference ConP *vs* ProbP were assessed by Independent-sample T-test. Data are expressed as means ± SEM.

### 3.2 Diet, energy intake and appetite

**Fig. 3:**
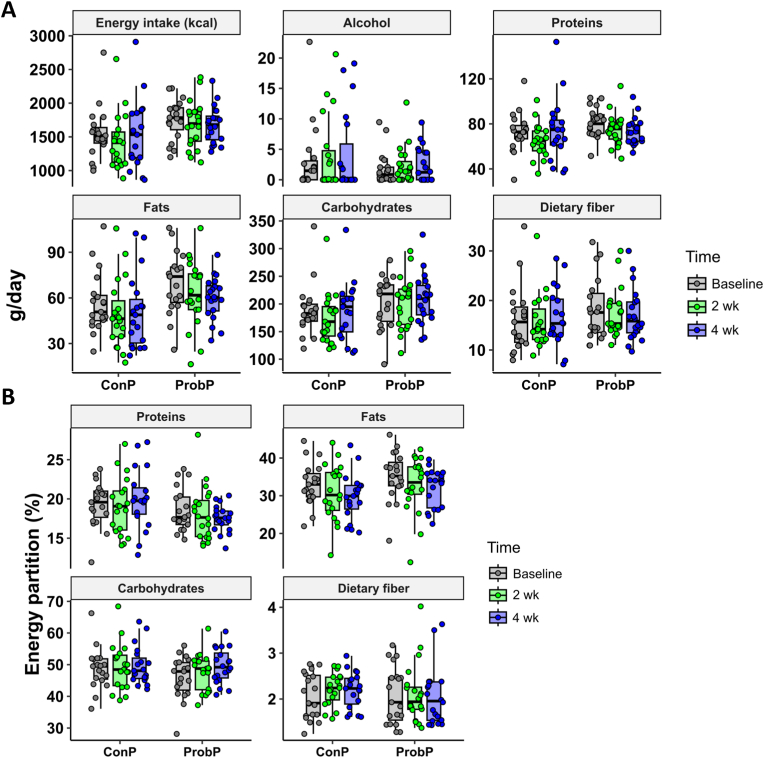
Daily energy intake and diet nutritional composition (A) and energy partition (B) assessed by 7 day-weighed food diaries self-recorded by participants over the run-in week and after 2 (2 wk) and 4 weeks (4 wk) of the intervention with the probiotic pasta (ProbP) and conventional pasta (ConP). Results are shown as mean ± SD. The box plots show the data distribution based on the first quartile, median and third quartile.

Fig. 3 shows the total energy intake and nutritional composition of the diets self-recorded by participants on the week before the intervention (baseline), after 2 and 4 weeks of intervention.

No between- and within-group differences were found in energy intake and nutritional composition over the study period.

The intake of the main food categories monitored over the study period is reported in Supplementary Table 3. No significant changes in the consumption of specific food groups were observed within the ConP and ProbP groups over the intervention, except for cheese consumption, which was lower after 4 weeks in the ProbP group compared to ConP, and yogurt intake, which increased after 4 weeks in ConP compared to ProbP.

Supplementary Fig. 2 shows the mean scores of hunger, fullness, and satiety sensations related to meals (breakfast, midmorning snack, before lunch, after lunch, afternoon snack, dinner, after-dinner snack) and across the weeks (baseline, 2 weeks, 4 weeks). No overall between-group difference was found in appetite sensations. ConP reported a significant lower hunger, higher fullness and satiety after the afternoon snack after 2 weeks compared to ProbP.

### 3.3 Gut permeability and gut microbiome

**Fig. 4:**
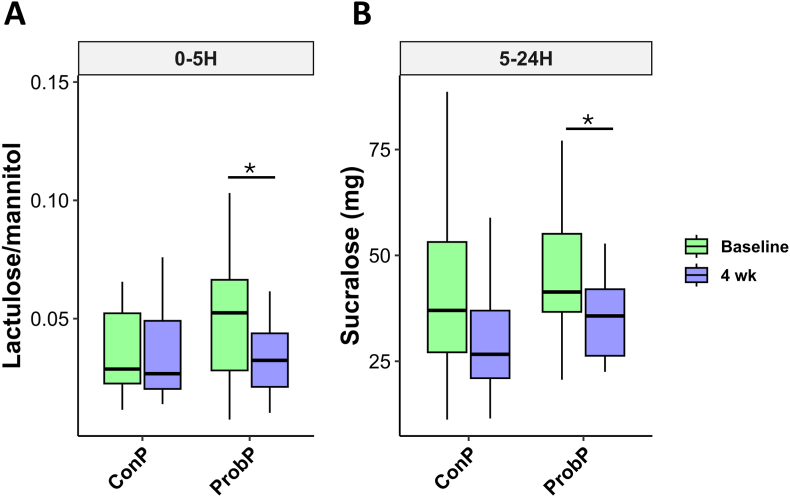
Concentrations of lactulose/mannitol in urines collected over the interval 0–5 h (A) and concentrations of sucralose in urines collected over the interval of 5–24 h (B) from participants during the gut permeability before (baseline) and after 4 weeks (4 wk) of consuming the conventional pasta (ConP) or probiotic pasta (ProbP). ∗p < 0.05 indicate significant differences between time points within groups by paired T-test. The box plots show the data distribution based on the first quartile, median and third quartile.

The 24-h urinary excretions of lactulose, mannitol, and sucralose after ingesting the sugar solution are shown in Fig. 4.

The ProbP group significantly reduced the lactulose to mannitol ratio at the 0–5h interval and sucralose excretion during the 5–24h interval after 4 weeks compared to baseline, while the ConP group did not show any significant variation. Furthermore, ProbP showed a trend toward a reduction of indoxyl sulfate (p-value = 0.07) after 4 weeks compared to baseline (Supplementary Fig. 3).

**Fig. 5:**
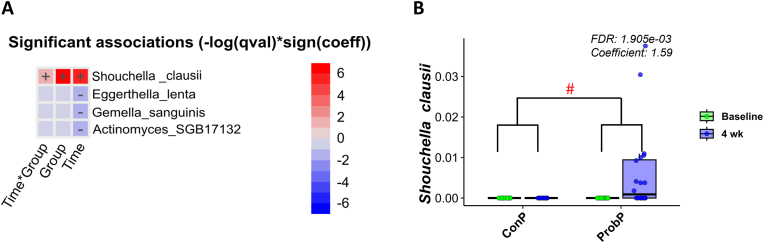
A) Heatmap showing the significant (q-value<0.05) effect of Time, Group and Time∗Group interaction upon *MaAsLin2* multivariate regression model. Positive associations (species with increased abundance) are shown in red while negative associations are shown in blue (species with increased abundance). B) *Shouchella clausii* relative abundance in participants who consumed the conventional pasta (ConP) or probiotic pasta (ProbP) at baseline and after 4 weeks (4 wk). The box plots show the data distribution based on the first quartile, median and third quartile.

With regards to the gut microbiome, only *S. clausii* increased its relative abundance (FDR <0.05, Maaslin2) after 4 weeks of ProbP consumption (Fig. 5a and b) compared to ConP. Interestingly, only 10 participants out of 20 in ProbP group showed increased *S. clausii* levels in the feces compared to the baseline, likely being more responsive to the ProbP compared to the others. No significant changes in Carbohydrate-Active Enzymes (CAZy) richness were observed during the nutritional intervention in either the ProbP or ConP groups (Supplementary Fig. 4).

### 3.4 Personalized response to the dietary intervention

**Fig. 6:**
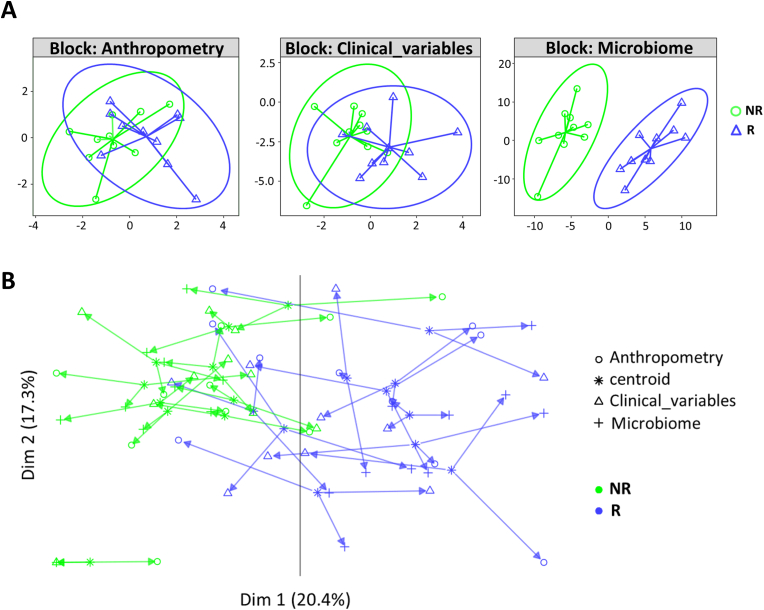
N-integrative supervised analysis of different types of datasets accounting for the within-variation (Δ_4weeks-baseline_). A) The DIABLO model for the discrimination of Responders (R) and Non responders (NR) groups is displayed as sample plot per single level (i.e. anthropometry, clinical variables and gut microbiome composition). B) Co-inertia analysis quantifying the co-variability between the three datasets. Shapes represent the projected coordinates of each subject. The centroid for a given sample between all datasets is indicated by the start of the arrow and the location of the same sample in each dataset by the tips of the arrows. The length of the arrow is proportional to the divergence between data from different blocks. The percentage of total explained variances describing the separation of the groups on the first two components are displayed on the x and y axis, respectively. Blue triangles, Responders (R) subjects. Green circles, Non-responders (NR) subjects. The AUC was 0.79 (p-value = 0.03) for anthropometry dataset, 0.87 (p-value = 0.005) for clinical variables dataset and, 0.99 (p-value = 0.0002) for gut microbiome when comparing the R and NR groups.

To test the hypothesis that an increased abundance of *S. clausii* in the gut might determine a personalized metabolic response to ProbP consumption, we subgrouped the ProbP participants into 'Responders' (R), including the 10 subjects showing an increase in *S. clausii* after 4 weeks, and 'Non-responders' (NR), including the remaining 10 subjects. By integrating anthropometry, clinical variables and gut microbiome datasets, we observed a separation of R and NR groups (by anthropometry, Hotelling T2 = 2.7, p = 0.09; by clinical variables, Hotelling T2 = 5.0, p = 0.02; by gut microbiome profile, Hotelling T2 = 103.6, p < 0.001) (Fig. 6a and b).

R and NR were homogeneous at baseline in terms of sex, age, and BMI (Table 3). However, R had significantly higher diastolic blood pressure (p-value = 0.006) and significantly lower defecation frequency over 24 h (p-value = 0.012) at baseline compared to NR. Although, on average, R had higher baseline systolic blood pressure, circulating total cholesterol, and LDL-cholesterol compared to NR, these differences did not reach statistical significance, likely due to high inter-individual variability or the small sample size.

**Table 3:** Metabolic parameters of Responders (R) and Non-responders (NR) volunteers at baseline and after 4 weeks (4 wk).

	NR (n = 10)		R (n = 10)		p-values
	Baseline	4wk	Baseline	4wk	Δ_(4wk-baseline)_
**Sex (n, F/M)**	5/5		4/6		
**Age**	36.5 ± 5.2		32.8 ± 3.6		0.28
**Waist/Hip**	0.8 ± 0.04	0.9 ± 0.04	0.9 ± 0.02	0.8 ± 0.02	0.11
**Body fat (%)**	27.3 ± 2.2	27.0 ± 2.2	28.3 ± 2.5	28.6 ± 2.3	0.29
**Body fat-free mass (%)**	51.5 ± 3.4	52.0 ± 3.2	53.3 ± 2.9	52.3 ± 2.9	0.17
**BMI (kg/m^2^)**	27.1 ± 0.8	27.2 ± 0.7	27.9 ± 1.0	27.7 ± 1.0**∗**	**0.049**
**Body weight (kg)**	78.7 ± 4.7	79.1 ± 4.5	81.6 ± 3.6	80.9 ± 3.5	**0.03**
**Systolic Blood Pressure (mmHg)**	112.2 ± 3.6	111.8 ± 3.8	118.1 ± 3.1	114.0 ± 3.9	0.19
**Diastolic Blood Pressure (mmHg)**	69.1 ± 2.3	69.9 ± 3.0	77.5 ± 2.0§	73.2 ± 2.3	**0.049**
**Physical activity level (MET min/wk)^a^**	2325.1 ± 628.2	1491.3 ± 273.1	1055.1 ± 211.4	2002.8 ± 740.5	0.32
**Glucose (mg/dL)**	90.9 ± 2.8	91.9 ± 2.0	93.1 ± 2.9	92.1 ± 2.6	0.51
**Insulin (μU/mL)**	7.4 ± 0.8	7.5 ± 1.1	8.4 ± 0.9	8.0 ± 1.1	0.76
**HOMA-index**	1.6 ± 0.2	1.7 ± 0.2	2.0 ± 0.2	1.8 ± 0.2	0.61
**Total cholesterol (mg/dL)**	144.1 ± 6.9	149.4 ± 6.6	153.5 ± 6.7	154.4 ± 3.6	0.59
**HDL-cholesterol (mg/dL)**	41.5 ± 3.4	44.7 ± 3.0	42.1 ± 2.0	48.2 ± 3.4∗	0.37
**LDL-cholesterol (mg/dL)**	85.1 ± 6.3	84.9 ± 5.7	93.7 ± 4.6	88.2 ± 2.6	0.30
**LDL-to-HDL ratio**	2.2 ± 0.3	2.0 ± 0.2	2.3 ± 0.2	1.9 ± 0.2∗	0.14
**Tryglicerides (mg/dL)**	87.9 ± 21.9	97.8 ± 18.6	89.0 ± 14.6	89.4 ± 13.6	0.52
**hs-CRP (mg/L)**	2.4 ± 1.1	2.3 ± 0.9	1.4 ± 0.6	1.1 ± 0.4	0.68
**King's stool chart score**	3.5 ± 0.3	3.6 ± 0.7	3.1 ± 0.4	3.0 ± 0.3	0.40
**Defecatory frequency in 24h (n)**	1.3 ± 0.2	1.3 ± 0.2	0.8 ± 0.1§	0.96 ± 0.2	0.18

∗p < 0.05 within-group difference assessed by paired T-test. Pairwise time points (Δ) difference NR *vs* R were assessed by Independent-sample T-test or Mann-Withney test. §p < 0.05 between-group difference at baseline NR *vs* R assessed by Independent-sample T-test. Data are expressed as means ± SEM.

Interestingly, R significantly increased circulating HDL-cholesterol while reducing BMI and the LDL-to-HDL ratio upon the intervention. Additionally, they showed a trend toward reduced body weight (p = 0.053) and LDL-cholesterol (p = 0.08) after 4 weeks compared to baseline. ProbP consumption was effective in reducing diastolic blood pressure and body weight in R compared to NR, as demonstrated by the between-group difference in pairwise time-point changes (Δ) (Table 3). No significant within-group differences were observed in the NR group. Neither group showed significant changes in physical activity over the study period.

Circulating endocannabinoids and N-acylethanolamines of R and NR groups monitored over the study period are shown in Supplementary Fig. 5. Specifically, compared to NR, participants in the R group showed significantly higher circulating concentrations of PEA, SEA, and 2-AG at baseline, as well as trends toward higher baseline concentration of AEA (p = 0.09) and lower OEA/PEA ratio (p = 0.06).

The analysis of the 7-day food diaries showed no significant differences in the macronutrient composition of the habitual diet at baseline between R and NR (Table 4). However, participants in the R group exhibited a trend toward a lower protein intake (p = 0.059) at baseline compared to NR.

**Table 4:** Daily energy intake and diet nutritional composition assessed by 7 day-weighed food diaries self-recorded by participants belonging to Responders (R) and Non-responders (NR) group over the run-in week and after 2 (2 wk) and 4 weeks (4 wk) of the intervention with the probiotic pasta (ProbP).

	NR (n = 10)			R (n = 10)			p-values	
	Baseline	2wk	4wk	Baseline	2wk	4wk	Δ_(2wk-baseline)_	Δ_(4wk-baseline)_
**Energy intake (kcal)**	1865.4 ± 71.7	1761.0 ± 116.1	1749.7 ± 98.2	1667.4 ± 108.5	1617.2 ± 103.2	1601.9 ± 70.4	0.99	0.97
**Alcohol (g)**	0.7 ± 0.4	1.4 ± 0.6	1.5 ± 0.7	2.8 ± 1.0	3.3 ± 1.2	3.3 ± 1.0	0.91	0.83
**Carbohydrates (g)**	223.2 ± 12.3	217.8 ± 13.8	229.1 ± 14.5	185.6 ± 15.5	185.3 ± 14.7	187.6 ± 11.1	0.99	0.58
**Dietary fiber/1000 kcal (g)**	10.1 ± 1.1	11.1 ± 0.8	10.3 ± 1.0	10.7 ± 1.0	10.0 ± 1.2	10.2 ± 1.1	0.45	0.70
**Dietary fiber (g)**	19.0 ± 2.4	19.3 ± 1.7	17.9 ± 1.9	17.6 ± 1.8	15.6 ± 1.1	16.2 ± 1.5	0.37	0.79
**Carbohydrates (%)**	45.9 ± 1.9	47.9 ± 1.1	50.4 ± 1.5	42.9 ± 2.2	44.6 ± 2.1	45.3 ± 1.5	0.99	0.58
**Lipids (g)**	74.5 ± 5.0	64.9 ± 6.6	60.3 ± 4.8	65.6 ± 7.6	61.1 ± 7.6	59 ± 4.2	0.81	0.52
**Lipids (%)**	36.6 ± 1.5	33.6 ± 1.8	31.7 ± 1.6∗	35.7 ± 2.6	33.9 ± 3.0	34.4 ± 1.6	0.86	0.32
**Proteins (%)**	19.2 ± 0.9	18.6 ± 0.9	17.8 ± 0.6	19.0 ± 0.9	18.7 ± 1.4	18.4 ± 0.6	0.43	0.43
**Proteins (g)**	87.0 ± 3.5	78.6 ± 5.2	75.5 ± 4.6	75.6 ± 4.3	71.2 ± 3.5	70.7 ± 2.7	0.65	0.45

∗p < 0.05 within-group difference assessed by repeated measure ANOVA. Pairwise time points (Δ) difference NR *vs* R were assessed by Independent-sample T-test or Mann-Whitney test. Data are expressed as means ± SEM.

No significant change in habitual diet macronutrient composition was observed over the study period, except for NR, who reduced the energy intake from lipids after 4 weeks compared to baseline. No between-group differences were registered for any of the macronutrients (Table 4 and Supplementary Fig. 6). Evaluating the dietary quantitative data at the level of food categories, participants in the R group consumed less fruit and more legumes at baseline compared to the NR group. Both R and NR significantly increased their consumption of refined grains (mainly due to pasta intake) after 2 and 4 weeks of nutritional intervention. No significant between-group changes in the consumption of specific food categories were observed over the intervention, except for oils and fats, which were consumed more by the R group after 4 weeks compared to NR. Food category intake is detailed in Supplementary Table 4.

### 3.5 Gut permeability of Responders and Non-responders

**Fig. 7:**
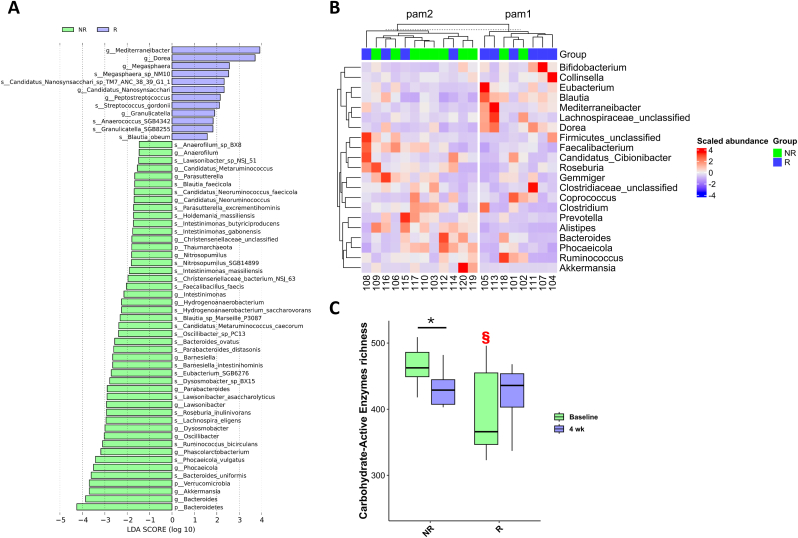
A) Linear discriminant analysis effect size (LEfSe) showing the differentially abundant taxa between Non-responders (NR; green) and Responders (R; blue) at baseline. The bacterial taxa shown exhibited a statistically significant change (p < 0.05) when the logarithmic linear discriminant analysis (LDA) score threshold was set to 2. The name of the taxon level is abbreviated as p-phylum, g-genus and s-species. B) Heatmap representing the genus-level composition of the gut microbiome at baseline in Non-responder (NR; green) and Responder (R; blue) groups (only taxa occurring in at least 50 % of the total subjects were included). Individuals were clustered by using the Partitioning Around Medoids (PAMs). C) Carbohydrate-Active Enzymes (CAZy) gene richness (expressed as the sum of the CAZy gene families) identified in Non-responders (NR) and Responders (R) at baseline and after 4 weeks (4 wk). §p < 0.05 between-group difference at baseline NR *vs* R assessed by Mann-Whitney test. ∗p < 0.05 within-group difference assessed by Mann-Whitney test.

A different gut microbiome at baseline between NR and R was found (Fig. 7a). R showed a higher abundance of *Dorea*, and *Mediterraneibacter* genera at baseline compared to NR, which conversely harboured in the gut higher abundance of *Bacteroides* and *Akkermansia* genera (Fig. 7a and b). Additionally, higher Carbohydrate-Active Enzymes (CAZy) richness was observed in NR compared to R at baseline, which decreased after 4 weeks of ProbP consumption compared to baseline (Fig. 7c).

**Fig. 8:**
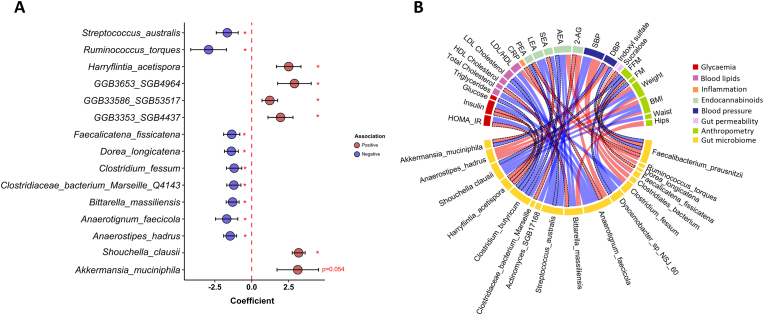
A) Species showing the significant (p-value<0.05) effect of Time upon *MaAsLin2* multivariate regression model within the Responders group. Baseline was put as the reference value. Positive associations (species with increased abundance upon 4 weeks) are shown in red while negative associations are shown in blue (species with increased abundance upon 4 weeks). B) Chord diagram showing Spearman's correlations among changes (pairwise time points difference 4 wk-baseline) in anthropometric and clinical variables with gut microbiome species that significantly varied after 4 weeks of ProbP consumption in NR and R groups. The line width represents the magnitude of Spearman's rho coefficient, with red indicating positive correlations and blue indicating negative correlations. Dotted black lines represents a statistical trend p-value<0.1 & >0.05. 2-AG, 2-Arachidonoylglicerol; AEA, Arachidonoylethanolamide; LEA, Linoylethanolamide; OEA, Oleoylethanolamide; PEA, Palmitoylethanolamide; SEA, Stearoylethanolamide; SBP, Systolic Blood Pressure; DBP, Diastolic blood pressure; FFM, Fat-free mass; FM, Fat mass; CRP, C-reactive protein.

R showed a significant increase of *Harryflintia acetispora* and a trend toward increased abundance of *Akkermansia muciniphila* (p = 0.054) after 4 weeks of ProbP consumption compared to the baseline, while decreasing the abundance of *Ruminococcus torques*, *Streptococcus australis*, *Faecalicatena fissicatena,* and *Dorea longicatena* (Fig. 8a). Conversely, the NR significantly reduced *Clostridium butyricum* and *Faecalibacterium prausnitzii* abundance upon 4 weeks of ProbP consumption compared to baseline (Supplementary Table 5). Gut microbial species that significantly varied after 4 weeks of ProbP consumption in NR and R groups were used to infer potential correlations with clinical and anthropometrical variable changes over the nutritional intervention study (Fig. 8b). The strongest correlations were shown by *Anaerotignum faecicola* changes which were negatively associated with insulin changes (Spearman's Rho = 0.6, p value = 0.002) and positively associated with plasma 2-AG changes (Spearman's Rho = 0.6, p value = 0.002). The changes in the abundance of CAZymes families in both R and NR is detailed in the Supplementary Table 6.

**Fig. 9:**
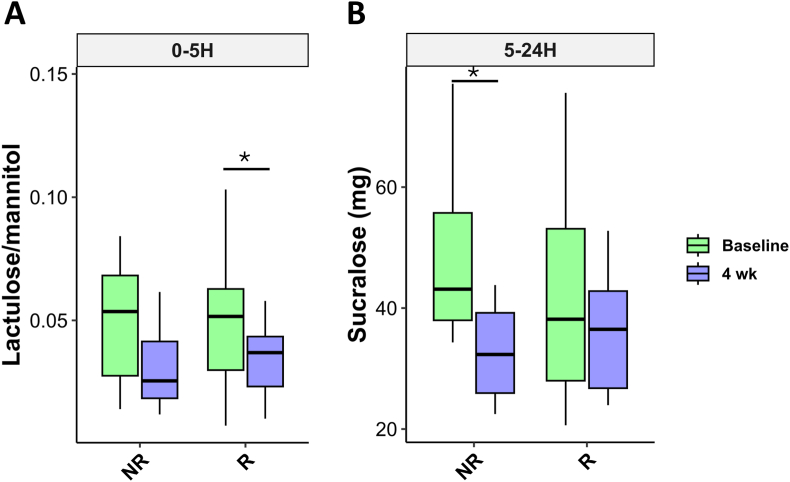
Urinary excretion of lactulose/mannitol during the interval of 0–5 h (A) and urinary excretion of sucralose during the interval of 5–24 h (B) of participants belonging to Responders (R) and Non-responders (NR) groups during the gut permeability test at baseline and after 4 weeks (4 wk). ∗p < 0.05 indicate significant differences between time points within groups by paired T-test. The box plots show the data distribution based on the first quartile, median and third quartile.

The 24-h urinary excretions of lactulose, mannitol, and sucralose after ingesting the sugar solution during the GPT are shown in Fig. 9. Results showed that after 4 weeks of ProbP consumption, the R group exhibited a significant reduction in urinary lactulose/mannitol excretion in the 0–5h interval compared to baseline, whereas the NR group showed a decrease in sucralose excretion in the 5–24h interval. These findings suggest that the metabolic improvements observed in the R group are unlikely to be solely attributable to changes in gut permeability.

Baseline urinary excretion of indoxyl sulfate was significantly higher in the R group compared to NR (Supplementary Fig. 7). Responders tendentially reduced the urinary excretion of indoxyl sulfate after 4-weeks of ProbP consumption (p = 0.07).

## 4 Discussion

### 4.1 Main findings

The most significant clinical effect of the probiotic pasta consumption was the increase in circulating HDL-cholesterol after four weeks, compared to baseline. Although improvements in HDL levels following probiotic supplementation have been reported inconsistently (López-Yerena et al., 2024; Momin et al., 2023), our findings align with those of previous interventions using spore-forming probiotics (Campbell et al., 2020; Angelino et al., 2019). This result was likely due to the improvement in gut permeability observed in participants consuming ProbP, as assessed by the reduced urinary excretion of lactulose/mannitol ratio and sucralose, compared with those consuming ConP (Mishra and Makharia, 2012; de Roos et al., 2017).

Impaired gut permeability is associated with overweight and obesity and is a causal factor in endotoxemia, including dyslipidemia (Ott et al., 2017; Violi et al., 2022). Several *in vitro* and *in vivo* studies have demonstrated that *S. clausii* can modulate the immune system, strengthen the mucosal barrier by improving intestinal permeability through enhanced mucin production, and produce short-chain fatty acids, which may further influence lipid metabolism (Dang et al., 2024; Acosta-Rodríguez-;Bueno et al., 2022). In contrast to previous RCTs in which probiotic supplementation did not improve gut barrier function, as assessed by urinary lactulose/mannitol ratio (Chaiyasut et al., 2021; Lee et al., 2014; Leber et al., 2012), our findings align with a study conducted in overweight elderly individuals, which reported a reduction in this ratio following 12 weeks of supplementation with a multi-strain probiotic *(Lactobacillus paracasei* HII01, *Bifidobacterium breve*, and *Bifidobacterium longum*; 5 × 10^10^ CFU/day) (Chaiyasut et al., 2022).

### 4.2 Colonization-permissive individuals show a personalized response to the dietary intervention

Despite variations in gut permeability in the ProbP group, no significant changes were observed in gut microbiome composition, except for *S. clausii* abundance, which increased at the end of the intervention in 10 out of 20 participants. Interestingly, this subset of responders showed a distinct baseline gut microbiome composition compared to those recalcitrant to *S. clausii* colonization. It is known that the responsiveness of the gut microbiome influences gut colonization ability of probiotic strains (Maldonado-;Gómez et al., 2016; Zmora et al., 2018; Montassier et al., 2021). For instance, a previous study showed that orally administered *B. longum* AH1206 persisted in only 30 % of the population, and those individuals had specific signatures in their gut microbiome and lower numbers of genes related to carbohydrate metabolism (Maldonado-;Gómez et al., 2016). Consistently, we observed that R exhibited a lower CAZy richness than NR. The baseline gut microbiota, due to competition for nutrients and adhesion sites, makes probiotic colonization highly personalized (Maldonado-;Gómez et al., 2016; Zmora et al., 2018; Montassier et al., 2021) and drives individualized response to nutritional interventions in terms of their impact on host's metabolism, as recently demonstrated with dietary fibers (Pihelgas et al., 2024). In our population, R showed higher baseline abundance of *Dorea* and *Mediterraneibacter* in the gut microbiome, which has been previously linked to host obesity (Abdugheni et al., 2022; Zeng et al., 2019). Conversely, the *Bacteroides* genus dominated in NR's gut, aligning with the higher habitual protein consumption compared to R. The *Bacteroides*-associated enterosignature, linked to high-protein and animal-fat diet, plays a central role in the resilience of Westernized gut microbiome (Frioux et al., 2023; Wu et al., 2011). Recently, individuals showing a gut ecosystem dominated by *Bacteroides* have been found less prone to shifts in gut microbiome composition following nutritional interventions (Pihelgas et al., 2024; Klimenko et al., 2022).

The higher blood pressure, along with higher circulating levels of PEA, SEA and 2-AG, as well as urinary excretion of indoxyl sulfate at baseline in R compared to NR, were also noteworthy. Increased visceral obesity and cardiometabolic risk factors have been previously associated with high circulating levels of endocannabinoids and their congeners (Côté et al., 2007; Engeli, 2008). Furthermore, the different baseline endocannabinoid tone may have driven the personalized response to ProbP consumption, as previously reported for a Mediterranean diet intervention (Tagliamonte et al., 2021). Indeed, we identified distinct shifts in both clinical variables and gut microbiome composition among R and NR after the intervention. Probiotic pasta consumption in R led to lipid profile changes that were consistent with findings previously observed in a subgroup of obese participants consuming symbiotic pasta enriched with *Bacillus coagulans* BC30 spores (Angelino et al., 2019). Specifically in the present study, the 14 % increase in HDL-cholesterol and the 17 % decrease in LDL-to-HDL ratio, a predictor of coronary atherosclerotic heart disease, might be clinically relevant (Sun et al., 2022). These changes, along with the reduced body weight and diastolic blood pressure in R, align with shifts in the gut microbiome profile. In addition to the increased *S. clausii* levels, there was an increase in SCFA-producing microorganisms, such as *Harryflintia acetispora* and *Akkermansia muciniphila* (Pessoa et al., 2023; Cani et al., 2022), and a reduction in microorganisms associated with inflammation and obesity, including *Ruminococcus torques*, *Streptococcus australis*, *Faecalicatena fissicatena*, and *Dorea longicatena* (Meijnikman et al., 2020; Companys et al., 2021; Chen et al., 2020; Schaus et al., 2024), along with the improvement in small intestine permeability. On the other hand, the reduced gut permeability in NR, which was not associated with metabolic benefits for the host, suggested that the presence of *S. clausii* in the gut of R might have played a key role in creating an environment that supported the aforementioned gut microbiome and metabolic changes, but only in R. Our identification of colonization-permissive and non-permissive individuals contributes to the growing body of evidence that probiotic colonization is not universal (Maldonado-Gómez et al., 2016; Zmora et al., 2018; Montassier et al., 2021). However, few clinical trials have stratified outcomes based on colonization status (DiMattia et al., 2024), highlighting a critical gap in understanding the personalized efficacy of probiotic interventions.

### 4.3 Strengths and limitations

This study has several notable strengths. First, it is the first RCT to evaluate the effects of consuming probiotic pasta enriched with *S. clausii* UBBC-07 spores, compared to conventional pasta, using a multi-system approach that examines nutrient metabolism, the endocannabinoid system, and the gut ecosystem, including gut permeability, all of which are known to play a key role in the development of cardiovascular disease. Second, the study involved participants at risk of cardiovascular disease due to overweight, obesity, and an unhealthy lifestyle, which allowed for the assessment of the potential preventive effects of probiotic pasta. Third, the intervention focused on incorporating a single food replacement within participants' habitual diets, offering new insights into the feasibility of probiotic-enriched foods as dietary interventions to improve health, an area with limited prior research. Finally, the study highlights a personalized response to *S. clausii* UBBC-07 colonization, influenced by individual microbiome characteristics, paving the way for more targeted probiotic interventions. Together, these strengths underscore the study's potential to inform future dietary strategies and guide the development of personalized functional foods aimed at preventing metabolic diseases and improving overall health.

However, the study also has some limitations. The first is the relatively short duration of the intervention, which may be insufficient to observe significant clinical changes, particularly in a population with an unhealthy lifestyle. The second limitation is the sample size, which may not be large enough to fully assess the impact of probiotic pasta consumption on the monitored variables in the subset of responders who experienced gut colonization by *S. clausii* after the intervention, compared to those who did not. Finally, although the responder group showed a statistically significant reduction in BMI after 4 weeks, the effect size was small and may be of limited clinical relevance. However, in the context of a short intervention, this modest change, alongside improvements in gut permeability and HDL-cholesterol, suggests early biological effects that may become more pronounced with longer treatment durations.

## 5 Conclusion

In conclusion, this randomized controlled trial demonstrated that consumption of probiotic pasta enriched with *S. clausii* UBBC-07 spores led to significant improvements in HDL-cholesterol and gut permeability compared to conventional pasta. Importantly, individual baseline microbiome profiles influenced both colonization and metabolic response, with colonization-permissive individuals (Responders) exhibiting notable reductions in LDL/HDL ratio, body weight, and diastolic blood pressure, accompanied by microbiome shifts characterized by increases in SCFA-producing taxa and decreases in obesity-associated microbes, mechanisms that likely underlie the observed metabolic benefits. In contrast, Non-responders showed improved gut permeability without systemic metabolic improvements, highlighting the critical role of colonization in mediating clinical outcomes. Altogether, these findings underscore the importance of individual microbiome profiling in the design of personalized functional foods that could effectively prevent metabolic disease through targeted modulation of the gut ecosystem.

## CRediT authorship contribution statement

**Silvia Tagliamonte:** Formal analysis, data curation, visualization, writing - Original Draft. **Roberta Barone Lumaga:** Investigation. **Francesca De Filippis:** Data curation, writing - Review & Editing. **Vincenzo Valentino:** Data curation. **Maria Aponte:** Resources. **Raffaele Romano:** Funding acquisition. **Paola Vitaglione:** Conceptualization, supervision, methodology, writing - Review & Editing.

## Funding

This research was funded by Italian 10.13039/100001239MISE (10.13039/501100007706Ministero dello Sviluppo Economico) within, the programme Horizon 2020 Fondo di crescita sostenibile, Sportello Agrifood 10.13039/501100021849Decreto Ministeriale March 05 2018, number F/200029/00/X45 “*Pasta funzionale probiotica*”- CUP B11B20000290005.

## Declaration of competing interest

The authors declare that they have no known competing financial interests or personal relationships that could have appeared to influence the work reported in this paper.

## Data Availability

The raw sequence reads generated in this study have been deposited in the Sequence Read Archive (SRA) of the NCBI under accession number PRJNA1198331. All software and databases used for analyses are publicly available for download. Other dataset supporting the current study are available from the corresponding author upon request.
