# Supplementary material for: Probiotic pasta consumption improves lipid metabolism and reduces gut permeability in overweight and obese adults: a randomized controlled trial
Source: Curr Res Food Sci. 2025 Oct 3;11:101215. doi: 10.1016/j.crfs.2025.101215 (PMC12538098; doi:10.1016/j.crfs.2025.101215)
Supplement: Multimedia component 1 [file mmc1.docx]

**SUPPLEMENTARY MATERIALS**

**Probiotic pasta consumption improves lipid metabolism and reduces gut permeability in overweight and obese adults: A randomized controlled trial**

Silvia Tagliamonte^a^, Roberta Barone Lumaga^a^, Francesca De Filippis^a,b^, Vincenzo Valentino^a^, Maria Aponte^a^, Raffaele Romano^a^, Paola Vitaglione*^a,b^

^a^ Department of Agricultural Sciences, University of Naples Federico II, 80055 Portici, Italy

^b^ Task Force on Microbiome Studies, University of Naples Federico II, 80134 Naples, Italy

***Corresponding author:**

Prof Paola Vitaglione, PhD

Department of Agricultural Sciences, University of Naples Federico II

Parco Gussone Ed. 84, Portici (NA), 80055, Italy

Telephone: +39 081 2539357

**Supplementary Table 1:** Calibration curve parameters for the quantification of Lactulose, Mannitol and sucralose in urine samples.

| **Compound** | **Linear range (μg/mL)** | **Regression equation** | **R^2^** |
| --- | --- | --- | --- |
| Lactulose | 0.25-5 | Y=0.036X-0.007 | 0.998 |
| Mannitol | 0.25-5 | Y=0.131X+0.046 | 0.996 |
| Sucralose | 0.5-10 | Y=0.022X+0.009 | 0.996 |

**Supplementary Table 2:** Calibration curve parameters for the quantification of N-acylethanolamines and endocannabinoids in plasma samples.

| **Compound** | **Linear range (ng/mL)** | **Regression equation** | **R^2^** |
| --- | --- | --- | --- |
| 2-Arachidonoylglicerol | 0.1-10 | Y=0.014X-0.000 | 0.996 |
| Arachidonoylethanolamide | 0.1-10 | Y=0.021X+0.001 | 0.997 |
| Linoylethanolamide | 0.5-25 | Y=0.028X+0.049 | 0.999 |
| Oleoylethanolamide | 0.1-10 | Y=0.058X-0.001 | 0.998 |
| Palmitoylethanolamide | 0.1-10 | Y=0.037X+0.010 | 0.006 |
| Stearoylethanolamide | 0.5-25 | Y=0.120X+0.000 | 0.999 |

**Supplementary Table 3:** Food category intake of participants who consumed conventional pasta (ConP) and probiotic pasta (ProbP) at baseline, 2 weeks (2 wk) and 4 weeks (4 wk).

|  | **ConP** | | | **ProbP** | | |  |  |
| --- | --- | --- | --- | --- | --- | --- | --- | --- |
|  | Baseline | 2 wk | 4 wk | Baseline | 2 wk | 4 wk | Δ_2wk-baseline_ | Δ_4wk-baseline_ |
| **Fresh Meat and Processed meat** | |  |  |  |  |  |  |  |
| Fresh meat | 32.29±6.75 | 38.41±8.20 | 35.94±8.78 | 29.97±7.28 | 36.82±8.57 | 39.23±6.90 | 0.91 | 0.66 |
| Processed meat | 29.49±7.13 | 22.02±5.26 | 23.91±5.23 | 27.82±5.83 | 19.18±3.63 | 23.19±4.40 | 0.87 | 0.62 |
| **Fishery** | 17.86±5.47 | 17.11±5.02 | 20.96±6.05 | 11.92±3.33 | 17.64±3.74 | 12.70±3.24 | 0.55 | 0.59 |
| **Eggs** | 0.90±0.90 | 1.43±1.02 | 2.26±1.28 | 0.90±0.90 | 0.86±0.86 | 0.00±0.00 | 1.00 | 0.23 |
| **Cereals** |  |  |  |  |  |  |  |  |
| Refined-grain products | 91.66±11.34 | 112.53±6.93* | 124.05±7.52* | 87.83±9.02 | 132.89±9.99* | 136.22±7.80* | 0.08 | 0.34 |
| Whole-grain products | 0.84±3.67 | 2.11±1.48 | 1.88±1.11 | 9.09±2.87 | 2.57±1.69 | 3.61±1.62 | 0.06 | 0.05 |
| **Milk & Dairy products** |  |  |  |  |  |  |  |  |
| Whole milk | 2.26±2.26 | 0.75±0.75 | 0.00±0.00 | 4.61±3.07 | 1.07±1.07 | 5.36±4.99 | 0.61 | 0.51 |
| Semi-skimmed milk | 11.73±8.11 | 3.46±3.46 | 3.87±2.81 | 42.24±21.62 | 43.93±20.32 | 34.77±19.48 | 0.15 | 0.87 |
| Cheese | 24.03±5.69 | 24.52±4.76 | 22.36±5.25 | 44.16±7.29 | 27.39±5.56 | 40.19±6.43 | **0.04** | 0.87 |
| Yogurt | 11.21±10.99 | 17.74±10.75 | 27.87±14.50 | 8.00±3.17 | 11.39±5.00 | 8.79±4.75 | 0.33 | **0.03** |
| **Fruits** |  |  |  |  |  |  |  |  |
| Fresh fruits | 97.18±21.26 | 95.24±23.30 | 107.22±23.89 | 89.05±19.10 | 102.89±26.09 | 87.82±13.36 | 0.36 | 0.84 |
| Nuts | 11.82±5.91 | 9.64±4.69 | 6.35±3.52 | 11.11±3.71 | 13.91±5.11 | 12.71±3.16 | 0.37 | 0.84 |
| **Vegetables** |  |  |  |  |  |  |  |  |
| Vegetables | 121.30±17.68 | 133.56±13.94 | 125.55±17.19 | 166.49±29.10 | 160.79±22.65 | 121.83±17.12 | 0.33 | 0.13 |
| Potatoes | 15.24±5.49 | 8.25±2.83 | 14.95±4.24 | 10.46±3.40 | 12.71±4.37 | 15.99±4.80 | 0.16 | 0.48 |
| **Legumes** | 19.91±9.71 | 14.30±3.10 | 14.35±2.92 | 3.95±1.63 | 9.64±2.73 | 8.67±3.99 | 0.36 | 0.40 |
| **Oils&Fats** | 4.18±1.61 | 6.14±2.29 | 6.18±2.44 | 5.80±1.82 | 6.86±1.82 | 6.74±1.87 | 0.87 | 0.83 |
| **Sweets and Snacks** | 59.41±8.58 | 45.01±10.82 | 42.45±8.31 | 48.83±9.08 | 54.99±8.37 | 53.85±8.90 | 0.53 | 0.31 |
| **Soft drinks** | 14.74±11.85 | 9.21±5.05 | 1.95±1.55 | 21.04±8.38 | 13.98±6.96 | 18.93±7.98 | 0.90 | 0.43 |
| **Alcoholic beverages** | 50.21±21.70 | 58.15±26.14 | 68.57±27.68 | 27.08±12.20 | 36.24±12.53 | 42.75±11.90 | 1.00 | 0.95 |

* p < 0.05 within-group difference assessed by repeated measure ANOVA. Pairwise time points (Δ) difference ConP *vs* ProbP were assessed by Independent-sample T-test or Mann-Withney test. Data are expressed as means ± SEM.

**Supplementary Table 4:** Food category intake of Responders (R) and Non responders (NR) volunteers at baseline, 2 weeks (2 wk) and 4 weeks (4 wk).

|  | **NR** | | | **R** | | |  |  |
| --- | --- | --- | --- | --- | --- | --- | --- | --- |
|  | Baseline | 2 wk | 4 wk | Baseline | 2 wk | 4 wk | Δ_2wk-baseline_ | Δ_4wk-baseline_ |
| **Meat** | |  |  |  |  |  |  |  |
| Fresh meat | 38.05±19.29 | 34.50±11.99 | 41.14±16.03 | 22.69±10.04 | 39.14±19.40 | 37.32±16.76 | 0.25 | 0.64 |
| Processed meat | 25.83±10.63 | 11.21±15.87 | 27.71±18.77 | 29.61±8.72 | 27.14±20.81 | 18.66±21.37 | 0.38 | 0.22 |
| **Fishery** | 11.97±6.79 | 9.29±7.52 | 13.87±10.79 | 10.45±5.81 | 20.71±11.99 | 7.26±7.76 | 0.13 | 0.9 |
| **Eggs** | 1.90±1.90 | 0.00±0.00 | 0.00±0.00 | 0.00±0.00 | 1.71±1.71 | 0.00±0.00 | 0.18 | 0.3 |
| **Cereals** |  |  |  |  |  |  |  |  |
| Refined-grain products | 101.57±14.65 | 149.36±15.58* | 147.26±13.51* | 75.46±10.07 | 116.43±10.88* | 125.19±6.88* | 0.73 | 0.78 |
| Whole-grain products | 9.03±3.92 | 0.86±0.86 | 3.79±2.04 | 75.46±10.07 | 116.43±10.88 | 125.19±6.88 | 0.52 | 0.95 |
| **Milk & Dairy products** | |  |  |  |  |  |  |  |
| Whole milk | 9.25±6.28 | 2.14±2.14 | 10.00±10.00 | 0.43±0.43 | 0.00±0.00 | 0.71±0.71 | 0.24 | 0.87 |
| Semi-skimmed milk | 39.97±30.96 | 32.86±24.10 | 39.55±26.41 | 44.29±31.76 | 55.00±33.71 | 30.00±30.00 | 0.52 | 0.26 |
| Cheese | 51.20±14.11 | 40.43±13.46 | 45.88±12.71 | 37.82±15.04 | 14.36±7.17 | 34.50±12.03 | 0.21 | 0.9 |
| Yogurt | 8.33±4.70 | 12.50±6.84 | 9.29±7.23 | 7.70±4.54 | 10.29±7.66 | 8.29±6.55 | 0.74 | 0.54 |
| **Fruits** |  |  |  |  |  |  |  |  |
| Fresh fruits | 128.19±31.89 | 126.07±46.38 | 96.21±19.54 | 53.81±16.75**§** | 79.71±24.57 | 79.43±18.88 | 0.5 | 0.11 |
| Nuts | 11.68±5.73 | 18.57±9.47 | 16.95±5.77 | 10.60±5.08 | 9.24±3.95 | 8.46±2.24 | 0.44 | 0.26 |
| **Vegetables** |  |  |  |  |  |  |  |  |
| Vegetables | 191.90±48.19 | 159.50±41.09 | 124.17±27.87 | 143.62±35.17 | 162.07±21.87 | 119.50±21.42 | 0.14 | 0.41 |
| Potatoes | 13.33±5.63 | 17.43±7.53 | 16.19±5.04 | 7.88±4.12 | 8.00±4.35 | 15.79±8.48 | 0.41 | 0.8 |
| **Legumes** | 0.48±0.48 | 9.14±5.48 | 5.62±4.40 | 7.07±4.52**§** | 10.14±7.70 | 11.71±12.13 | 0.43 | 0.96 |
| **Oils&Fats** | 5.39±2.72 | 1.57±0.94 | 3.14±1.46 | 6.18±2.58 | 12.14±2.63 | 10.34±3.13 | **0.02** | 0.12 |
| **Sweets and Snacks** | 45.65±9.08 | 47.90±10.75 | 53.55±11.56 | 51.69±8.87 | 62.09±13.00 | 54.15±14.17 | 0.43 | 0.85 |
| **Soft drinks** | 20.29±14.09 | 15.24±12.78 | 4.43±4.43 | 21.71±10.37 | 12.86±7.28 | 33.42±14.24 | 0.58 | 0.23 |
| **Alcoholic beverages** | 5.00±5.00 | 27.46±15.22 | 19.50±10.45 | 46.95±21.28 | 44.14±19.91 | 66.00±19.21 | 0.22 | 0.93 |

* p < 0.05 within-group difference assessed by repeated measure ANOVA. Pairwise time points (Δ) difference NR *vs* R were assessed by Independent-sample T-test or Mann-Withney test. § p < 0.05 between-group difference at baseline NR *vs* R assessed by Independent-sample T-test. Data are expressed as means ± SEM.

**Supplementary Table 5:** Species showing the significant (p-value<0.05) effect of Time upon *MaAsLin2* multivariate regression model within Non responders (NR). Baseline was put as the reference value. Positive associations represent species with increased abundance upon 4 weeks while negative associations represent species with increased abundance upon 4 weeks.

| **Feature** | coef | stderr | pval |
| --- | --- | --- | --- |
| Clostridium_butyricum | -0.00814 | 0.002753 | 0.016008 |
| GGB9760_SGB15373 | -0.06685 | 0.025033 | 0.025596 |
| Faecalibacterium_prausnitzii | -1.25168 | 0.495537 | 0.032453 |
| Clostridiales_bacterium | -0.00923 | 0.003738 | 0.035634 |
| GGB9707_SGB15229 | -0.03234 | 0.013126 | 0.035933 |
| GGB9559_SGB14969 | -0.02372 | 0.00977 | 0.038091 |
| GGB3570_SGB4777 | -0.02328 | 0.009928 | 0.043668 |
| GGB80011_SGB15265 | 0.802611 | 0.345174 | 0.045098 |
| Dysosmobacter_sp_NSJ_60 | -0.01436 | 0.006217 | 0.046252 |
| Actinomyces_SGB17168 | -0.00582 | 0.002541 | 0.04767 |

**Supplementary Table 6:** Carbohydrates-Active enZYmes (CAZy)showing the significant (p-value<0.05) effect of Time upon *MaAsLin2* multivariate regression model within Non responders (NR). Baseline was put as the reference value. Positive associations represent species with increased abundance upon 4 weeks while negative associations represent species with increased abundance upon 4 weeks.

| **Feature** | Group | coef | stderr | pval |
| --- | --- | --- | --- | --- |
| CBM54 | NR | -0.72801 | 0.206721 | 0.006498 |
| GT66 | NR | 1.426816 | 0.513671 | 0.021482 |
| GH170 | NR | -0.53672 | 0.209227 | 0.030424 |
| PL33_1 | NR | -0.30165 | 0.118659 | 0.031597 |
| CE12 | NR | 0.219057 | 0.086937 | 0.032784 |
| CBM66.PL11 | NR | 1.06757 | 0.431703 | 0.0354 |
| GH91 | NR | -0.47183 | 0.202054 | 0.044369 |
| GT2.GT8 | NR | 0.683723 | 0.300407 | 0.048882 |
| CBM48.CE0 | NR | -0.59553 | 0.262492 | 0.049463 |
| CBM5.GH18 | R | -1.07647 | 0.240785 | 0.001553 |
| CBM56.GH81 | R | 2.606982 | 0.656886 | 0.003261 |
| CBM47 | R | 4.858408 | 1.291937 | 0.004481 |
| GH4 | R | -0.47038 | 0.163091 | 0.009878 |
| GH102 | R | -0.28884 | 0.092758 | 0.01244 |
| CBM32.GH123 | R | 4.423478 | 1.441179 | 0.013368 |
| GH43_26 | R | -0.51977 | 0.177737 | 0.016911 |
| GH13_12 | R | -1.27184 | 0.452414 | 0.020336 |
| GH13_29 | R | -0.34541 | 0.123854 | 0.021094 |
| GH10 | R | -0.35048 | 0.126536 | 0.021759 |
| CBM51 | R | 1.577521 | 0.646521 | 0.025259 |
| GH136 | R | -0.41413 | 0.156769 | 0.026842 |
| CBM0.CBM32.GH89 | R | -1.33946 | 0.507278 | 0.026892 |
| PL1.PL9_1 | R | 2.607723 | 1.004219 | 0.028891 |
| CBM32.GH89 | R | -1.1517 | 0.44755 | 0.030023 |
| CBM46.GH5_4 | R | -2.17882 | 0.852931 | 0.030965 |
| GH36 | R | -0.15217 | 0.05973 | 0.031318 |
| CBM32.GH36 | R | -0.91814 | 0.362089 | 0.031936 |
| GH43_10.GH5_21 | R | 2.749965 | 1.101023 | 0.033993 |
| GH130_5 | R | 1.733808 | 0.694195 | 0.033996 |
| GH1 | R | -0.31433 | 0.137282 | 0.034341 |
| CBM32.GH81 | R | -1.46272 | 0.589706 | 0.034967 |
| GH13_4 | R | -0.63963 | 0.257943 | 0.035006 |
| CBM50.CE4 | R | -1.5155 | 0.626718 | 0.038728 |
| CE8.PL1_2 | R | 1.468753 | 0.610048 | 0.039404 |
| GH5_26 | R | 2.039683 | 0.849248 | 0.039783 |
| GH64 | R | -1.1433 | 0.477418 | 0.040242 |
| GH112 | R | -0.31433 | 0.131613 | 0.04067 |
| CBM91.GH43_11 | R | -1.10245 | 0.464557 | 0.041695 |
| CBM72 | R | 1.476603 | 0.629607 | 0.043641 |
| CE9 | R | -0.24637 | 0.105864 | 0.044951 |
| CBM51.GH110 | R | 3.910901 | 1.684843 | 0.045395 |
| CBM66.GH32 | R | -1.02099 | 0.440896 | 0.045805 |

**
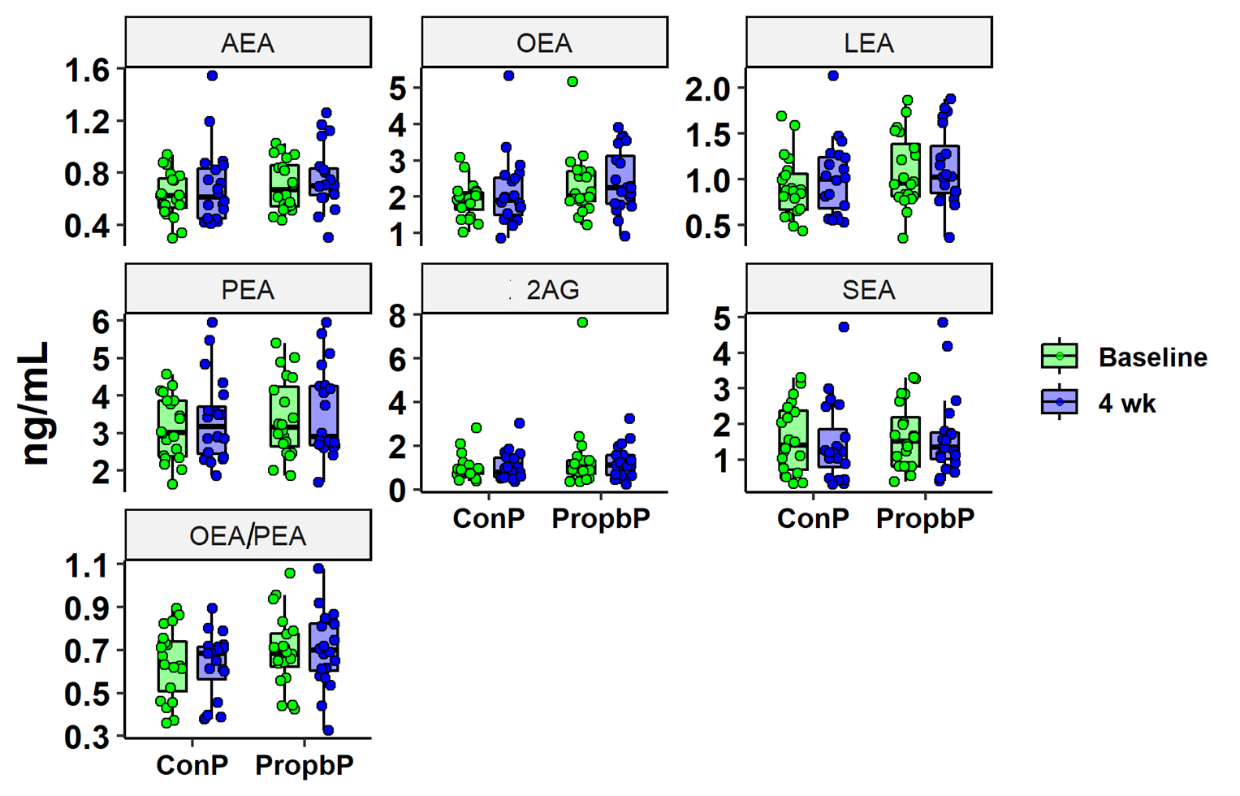
**

**Supplementary Fig. 1:** Plasma Endocannabinoids and N-acylethanolamines of participants who consumed conventional pasta (ConP) and probiotic pasta (ProbP) at baseline (0 wk) and 4 weeks (4 wk). 2-AG, 2-Arachidonoylglicerol; AEA, Arachidonoylethanolamide; LEA, Linoylethanolamide; OEA, Oleoylethanolamide; PEA, Palmitoylethanolamide; SEA, Stearoylethanolamide.


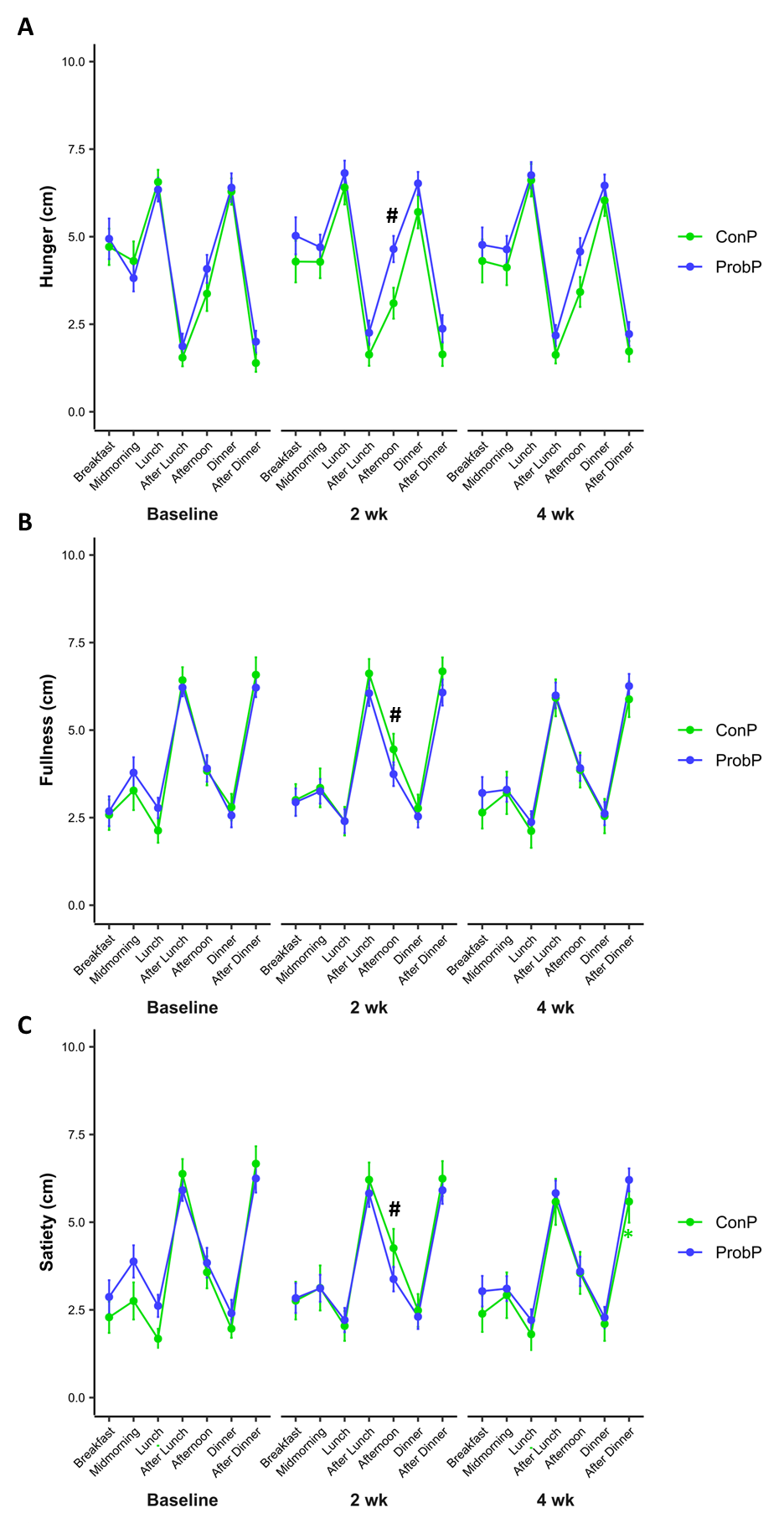


**Supplementary Fig. 2:** Hunger (A), fullness (B), and satiety (C) ratings (mean ±SEM) self-recorded by participants in the ConP and ProbP group before breakfast, morning snack, before lunch, after lunch, afternoon snack, before dinner, and after dinner snack for 7 d during the run-in and every intervention week. * p < 0.05 within-group difference vs baseline; # p < 0.05 between-group difference ProbP vs ConP  assessed by ANOVA repeated measures after controlling for baseline.


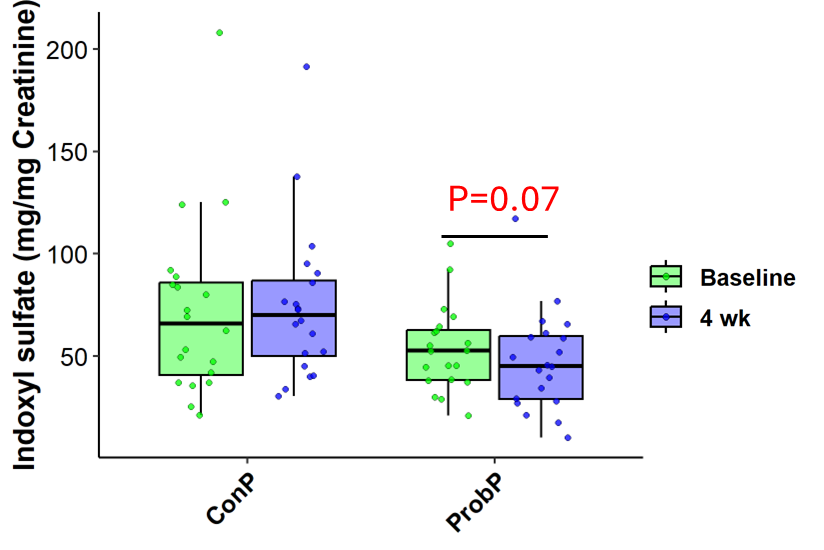


**Supplementary Fig. 3:** Urinary excretion of indoxyl sulfate of participants who consumed conventional pasta (ConP) and probiotic pasta (ProbP) at baseline and after 4 weeks (4 wk).


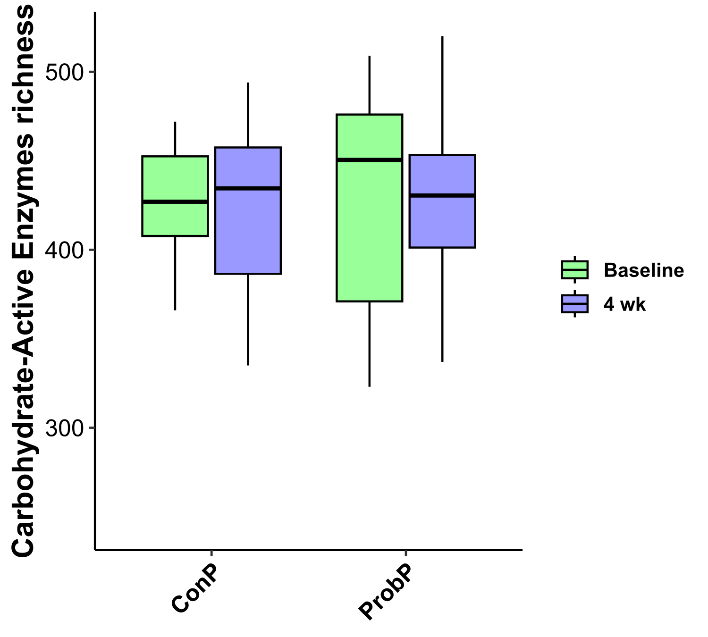


**Supplementary Fig. 4:** Sum of the microbial Carbohydrate-Active Enzymes (CAZy) families identified in participants consuming the conventional pasta (ConP) or probiotic pasta (ProbP) at baseline and after 4 weeks (4 wk).


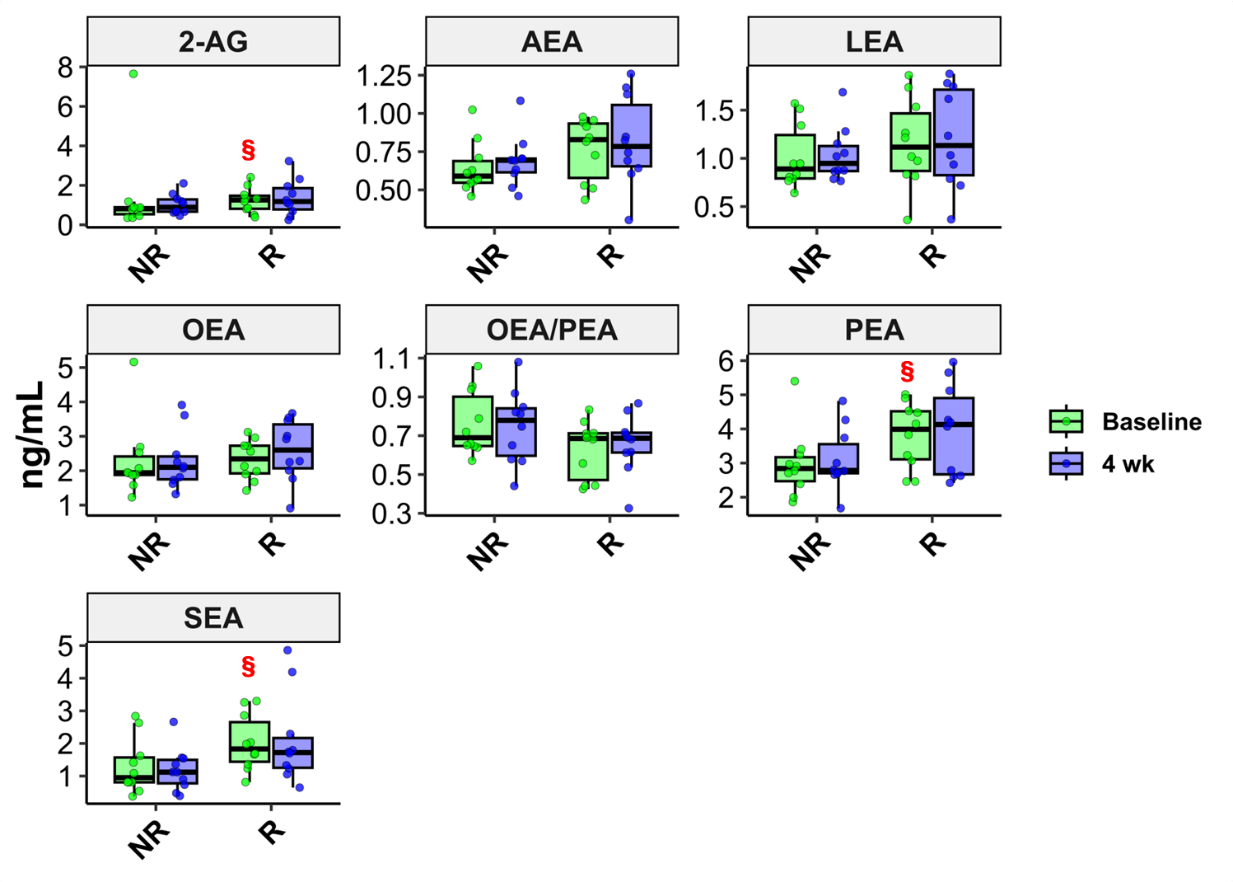


**Supplementary Fig. 5:** Plasma Endocannabinoids and N-acylethanolamines of participants from Responders (R) and Non-responders (NR) groups at baseline (0 wk) and after 4 weeks (4 wk). § p < 0.05 between-group difference at baseline NR *vs* R assessed by Independent-sample T-test.  2-AG, 2-Arachidonoylglicerol; AEA, Arachidonoylethanolamide; LEA, Linoylethanolamide; OEA, Oleoylethanolamide; PEA, Palmitoylethanolamide; SEA, Stearoylethanolamide.


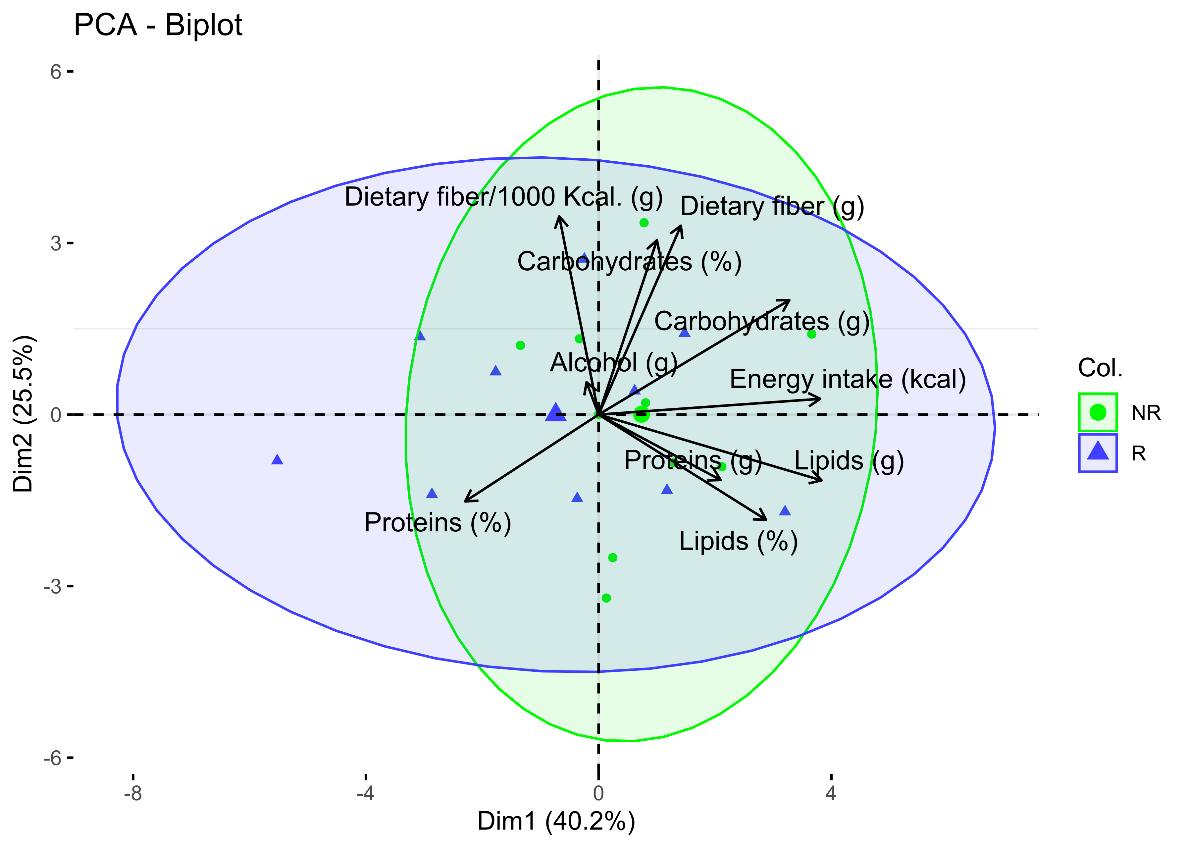


**Supplementary Fig. 6:** Principal component analysis biplot based on habitual macronutrients intake at baseline in Responder (R; Blue) and Non-responders (NR; Green). Between group distances were assessed by computing Mahalanobis distance and Hotelling’s T2 test (T2 = 1.1882, p-value = 0.3288).


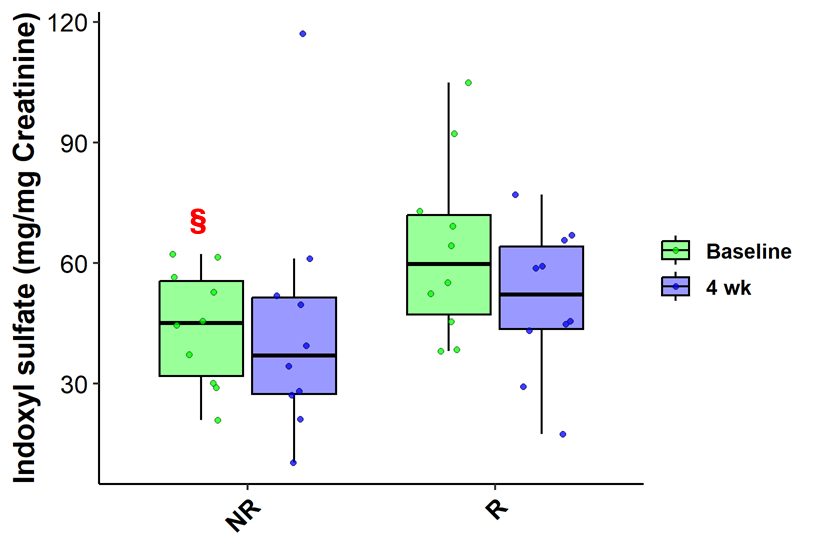


**Supplementary Fig. 7:** Urinary excretion of indoxyl sulfate of participants from Responders (R) and Non-responders (NR) groups at baseline and after 4 weeks (4 wk).
